# A transcriptional sketch of a primary human breast cancer by 454 deep sequencing

**DOI:** 10.1186/1471-2164-10-163

**Published:** 2009-04-20

**Authors:** Alessandro Guffanti, Michele Iacono, Paride Pelucchi, Namshin Kim, Giulia Soldà, Larry J Croft, Ryan J Taft, Ermanno Rizzi, Marjan Askarian-Amiri, Raoul J Bonnal, Maurizio Callari, Flavio Mignone, Graziano Pesole, Giovanni Bertalot, Luigi Rossi Bernardi, Alberto Albertini, Christopher Lee, John S Mattick, Ileana Zucchi, Gianluca De Bellis

**Affiliations:** 1 Institute of Biomedical Technologies, National Research Council, Milan, Italy; 2 Current address: Genomnia srl, via Nerviano, 31 – 20020 Lainate, Milano, Italy; 3 Department of Biochemistry and Molecular Biology, University of California Los Angeles, CA, USA; 4 Current address: Korean Bioinformation Center, Korea Research Institute of Bioscience and Biotechnology, 52 Eoeun-dong, Yuseong-gu, Daejeon, 305-806, South Korea; 5 Department of Biology and Genetics for Medical Sciences, University of Milan, Milan, Italy; 6 ARC Special Research Centre for Functional and Applied Genomics, Institute for Molecular Bioscience, University of Queensland, St Lucia, QLD 4072, Australia; 7 Translational Research Unit, Department of Experimental Oncology, Istituto Nazionale Tumori, Milan, Italy; 8 Faculty of Pharmacological Sciences, University of Milan, Milan, Italy; 9 Department of Biochemistry and Molecular Biology, University of Bari, Bari, Italy; 10 Division of Pathology and Laboratory Medicine, European Institute of Oncology, Milan, Italy; 11 Current address: Department of Pathology, Desenzano sul Garda Hospital, Leno, Italy; 12 Science and Technology Pole, Istituto di Ricovero e Cura a Carattere Scientifico MultiMedica, Milan, Italy

## Abstract

**Background:**

The cancer transcriptome is difficult to explore due to the heterogeneity of quantitative and qualitative changes in gene expression linked to the disease status. An increasing number of "unconventional" transcripts, such as novel isoforms, non-coding RNAs, somatic gene fusions and deletions have been associated with the tumoral state. Massively parallel sequencing techniques provide a framework for exploring the transcriptional complexity inherent to cancer with a limited laboratory and financial effort. We developed a deep sequencing and bioinformatics analysis protocol to investigate the molecular composition of a breast cancer poly(A)^+ ^transcriptome. This method utilizes a cDNA library normalization step to diminish the representation of highly expressed transcripts and biology-oriented bioinformatic analyses to facilitate detection of rare and novel transcripts.

**Results:**

We analyzed over 132,000 Roche 454 high-confidence deep sequencing reads from a primary human lobular breast cancer tissue specimen, and detected a range of unusual transcriptional events that were subsequently validated by RT-PCR in additional eight primary human breast cancer samples. We identified and validated one deletion, two novel ncRNAs (one intergenic and one intragenic), ten previously unknown or rare transcript isoforms and a novel gene fusion specific to a single primary tissue sample. We also explored the non-protein-coding portion of the breast cancer transcriptome, identifying thousands of novel non-coding transcripts and more than three hundred reads corresponding to the non-coding RNA *MALAT1*, which is highly expressed in many human carcinomas.

**Conclusion:**

Our results demonstrate that combining 454 deep sequencing with a normalization step and careful bioinformatic analysis facilitates the discovery and quantification of rare transcripts or ncRNAs, and can be used as a qualitative tool to characterize transcriptome complexity, revealing many hitherto unknown transcripts, splice isoforms, gene fusion events and ncRNAs, even at a relatively low sequence sampling.

## Background

The classic image of the mammalian transcriptome is composed of a large assembly of spliced mRNAs, each structured with a capped 5' end, a 5' untranslated region, a coding sequence, a 3' untranslated region and a polyA tail, together with a relatively well-defined set of non-protein-coding RNAs with different functions (ribosomal, transfer, spliceosomal and small nucleolar RNAs), with most of the genome thought to be genetically inert. Transcriptome sequencing and annotation initiatives have challenged this view by discovering that most of the genome is actively transcribed to yield complex patterns of interlaced and overlapping transcripts, including tens of thousands long (>200 nt) non-protein-coding RNAs (ncRNA) [1-3].

Non-coding RNAs (ncRNAs) have emerged as a diverse and important class of functional transcripts, accounting for approximately the 1.5% of the transcriptional output of mammalian genomes [4,5]. The regulatory role of these molecules has been clearly established for some species such as microRNAs (miRNAs) or small nucleolar RNAs (snoRNAs) [6,7]. In addition, although most have not yet been studied, many of the observed long 'mRNA-like' ncRNAs are differentially expressed and developmentally regulated, and increasing numbers are being shown to function in a range of processes in cell and developmental biology [8-13].

Compared to wild-type, the cancer cell transcriptome is grossly altered. Microarray studies have revealed a host of aberrations (*i.e. *drastic changes in expression levels of specific transcripts), and recent RNA-seq studies have identified a set of cancer-specific transcripts and transcriptional variants in tissues and cell lines [14-17]. Common alterations found in tumors are gene fusions and aberrant splicing isoforms [18,19]. Although prevalent in blood tumors, gene fusions occur in all malignancies, and they account for 20% of human cancer morbidity [20]. Alternative splicing is often deregulated in cancer, probably as a consequence of quantitative alterations in the levels of expression of splicing regulators [21]; however, many examples of cancer-specific gene isoforms (*CD44*, *BRCA1*, survivin etc), whose expression seem to correlate with the disease, have been described in literature [22].

A link between ncRNAs and cancer is becoming increasingly evident. For example, two ncRNAs, *PCGEM *and *DD3*, are significantly over expressed in prostate cancer, *HULC *expression is significantly associated with hepatocellular carcinoma [23] and *MALAT1 *is known to be over expressed in several human carcinomas [24-26]. Additionally, genes encoding hundreds of highly conserved ncRNAs are altered in a significant percentage of leukaemia and carcinomas [27].

To explore this complexity, we employed the Roche 454 deep sequencing technology [28] and biology-oriented sequence analysis techniques to obtain a transcriptional snapshot of a normalized primary breast cancer cDNA library. Our approach is largely qualitative, aiming at the identification of transcriptional events associated with the cancer phenotype. These included gene fusions, gene deletions, rare or aberrant transcriptional isoforms, ncRNAs, and transcripts of unknown function (TUF); a subset of interesting transcripts was validated using RT-PCR on the RNA obtained from the original breast cancer sample as well as from other eight carcinomas with the same histotype. Globally, our results demonstrate that direct pyrosequencing of a normalized human cDNA library coupled with bioinformatic analysis complements quantitative investigations of gene expression by providing an accurate qualitative picture of a complex transcriptome, potentially unraveling tissue or disease-specific transcriptional events.

## Methods

### cDNA library preparation, emulsion PCR and pyrosequencing

Polyadenylated RNA was isolated from a breast invasive tumor sample (*in situ *lobular carcinoma, bilateral, with elevated mitotic and proliferative index, G3, Tamoxifen treated, identified by the code 1360), having a purity of 85–90%. cDNA was synthesized using Super SMART™ PCR cDNA Synthesis Kit (Clontech, Mountain View, CA). Prior informed consent for the research use of biological material from surgery was obtained for this sample. The ethics committee of the Institute for Biomedical Technologies – National Research Council approved the use of this biological sample for the study presented here. After reverse transcription, the cDNA library was normalized to obtain an equilibrated mix of low and high abundance mRNAs using Kamchatka crab double-strand nuclease (DSN) [29], as described in Additional file 1.

2.1 μg of normalized double stranded cDNA was sheared by nitrogen nebulization following the manufacturer's instruction (Roche, Basel, Switzerland). Ligation of the nebulized sample to specific adaptors and preparation of the single strand libraries (sstDNA) was performed as previously described [28]. After purification, nebulized sstDNA preparation was quantitated by RiboGreen RNA Quantitation Kit (Invitrogen Inc., Carlsbad, California). Quality was assessed using an Agilent Bioanalyzer. All purification steps were performed using MinElute PCR Purification Kit (Qiagen, Hilden, Germany).

The sstDNA library was then amplified by emulsion PCR performed in water-in-oil microvescicles. Each PCR reaction was recovered by propanol emulsion breaking and buffer washing and enriched for positive reaction beads. The beads were then washed; the primers were annealed and then counted using the Multisizer™ 3 Coulter Counter (Beckman Coulter, Inc. Fullerton, CA, USA). The kits for DNA fragmentation, polishing, capture on beads, emulsion PCR and sequencing were purchased from Roche Diagnostics. Samples were loaded onto 70x75 PicoTiterPlate (PTP) and inserted in the 454 – Roche GS 20 Genome Sequencer for the pyrosequencing reaction.

### Sequence redundancy reduction

Sequence reads were extracted from the raw pyrosequencing data following the manufacturer's technical documentation. The technical redundancy in the dataset (perfect sequence duplication) was removed using the NCBI nrdb program, included in the downloadable Blast suite . After mapping the remaining reads to the genome, we employed a second sequence redundancy reduction step for the analyses investigating the overlap between our reads and genomic features such as ENCODE regions, ncRNAs or genes. For this purpose, we used the CleanUp Algorithm [30] to generate a new non-redundant dataset, using stringent cut-off parameters (similarity > 98%, coverage threshold > 98%). We used the Cap3 assembler [31] to perform all the transcript assemblies.

### Mapping to the transcriptome and genome

A detailed description of the bioinformatics methods used in this part of the work can be found in Additional file 1. All the database searches against known transcripts (such as ESTs) were performed using the NCBI BlastN program. Non-redundant sequence reads were compared with the human genome using Blat [32]. All human full-length transcripts annotated in UCSC database (all_mrna Table, all Human mRNAs from GenBank, human genome release hg18, March 2006) [33] were used as reference set for the classification. We defined a read as 'spliced' when mapping to a chromosome with a coverage > = 95% in at least two parts separated by a gap > = 50 nt. We classified a read as 'intragenic' when mapping at least partially within a known gene (either in an exonic or intronic region), otherwise it was classified as 'intergenic'. Additional criteria were used to build an 'exon-oriented' read classification.

A collection of Conserved Sequence Tags (CSTs) [34,35], obtained by a full-genome comparison of human and mouse genomes, was compared to the genome mappings of the cDNA reads, excluding reads located within known exons, to evaluate both conservation and coding propensity.

### Bioinformatic identification of cancer-specific splice sites and fusion/deletion transcripts

The details of the bioinformatics strategy used for detection of gene fusions and deletions is described in detail in Additional file 1. Briefly, we first detected alignments (using reads at least 50-bp long) corresponding to putative chromosomal rearrangements and then identified putative translocation-mediated interchromosomal fusion transcripts by comparing the gene direction at the predicted breakpoints with known exon boundaries. Using a similar procedure, we identified intragenic deletion events. Predictions were compared with data from the chimerDB database [18].

To analyze cancer-associated splicing events we used the ASAP II database [19], which catalogues validated cancer-associated isoforms curated from EST sequencing data. We identified deep-sequencing reads with high-quality alignments and at least one splice site, and compared them with 273 high-confidence cancer-specific splice sites (LOD > = 3) from 198 genes in ASAP II database.

### Analysis of non-protein coding transcripts

The breast cancer cDNA library reads were aligned to UCSC Known Genes FastA sequences (human genome release hg18, 260.731 entries) using BLAST, and were classified on the basis of their genomic location. The conservation profile of non-exonic reads was assessed using the UCSC PhastCons17way conservation score. A total of four different datasets were generated: intronic, extragenic, desert conserved and desert non-conserved. These datasets were subsequently cross referenced against CRITICA ncRNA predictions [12], a subset of RNAdb [36], and NONCODE [37]. Details of these bioinformatic analyses are available in Additional file 1. In addition, we assessed the overlap between cDNA reads and the ENCODE project annotation of novel transcribed region of unknown function [38] by intersecting high-quality genome-wide mappings with the genomic coordinates of the encodeRna Table at UCSC.

### Biological validation of selected transcripts

Validation was performed by direct sequencing of the cDNA library and RT-PCR. We used RNA obtained from the original lobular breast cancer sample and from other eight tumors and performed RT-PCR using an oligo (dT) primer and SuperScriptTM II Reverse Transcriptase (Invitrogen Inc., Carlsbad, California) according to manufacturer's instructions. For fusion transcripts we sequenced individual PCR products after cloning them into the pCR^®^II-TOPO TA vector (Invitrogen Inc., Carlsbad, California). Additional file five lists all the PCR primers and their annealing temperatures, together with the results of all validations experiments. Since we were investigating rare transcripts detected from a normalized cDNA library, we reasoned that RNA extracted directly from primary samples could be the best source of genetic material for validation.

## Results and discussion

### Assessment of the cDNA library normalization before and after deep sequencing

**Figure 1:**
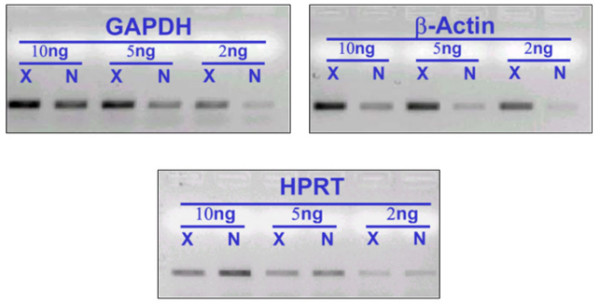
**Assessment of the cDNA library normalization before sequencing**. RT-PCR amplification of three reference genes, each expressed constitutively but with different abundance in the cell, to test the normalization of the cDNA library before sequencing. X = control before normalization. N = normalized library.

Aiming to detect rarely expressed transcripts, we complemented the standard deep-sequencing protocol with a normalization step. Reference genes, which are often referred to as 'housekeeping genes', are frequently used to normalize mRNA levels between different samples [39]. In order to assess the success of the cDNA library normalization procedure before sequencing, PCR amplifications with selected probes corresponding to reference genes were performed. Three reference genes with different expression levels were chosen for the analysis (Additional file 1). A visual inspection of the amplification bands in Figure 1 confirms that the normalization procedure decreased the level of highly expressed transcripts and increased the strength of the bands corresponding to low-level transcripts. For example, the expression of *GAPDH *(Glyceraldeide-3-phosphate dehydrogenase) is reduced and the expression of weakly expressed *HPRT1 *(Hypoxanthine phosphoribosyltransferase 1) is increased.

To assess whether the library normalization was also reflected in the 454 sequence output, we counted the reads that could be unequivocally associated with *ACTB *(Beta-actin), *GAPDH *and *HPRT *and compared them with the abundance of these transcripts (from the same tissue and pathological state as our experimental sample) in a public EST library (see related Methods in Additional file 1). We applied a well-established statistical test for assessing significant differences in digital gene expression profiles [40] and found that that the sequence sampling reflects the normalization of the cDNA library, even at the relatively shallow depth of sequencing accomplished with 454 (Table 1).

**Table 1:** Assessment of the cDNA library normalization by sequence count

**Reference Gene^1^**	**454 Reads mapped to the genome (194,806)**	**UniGene ESTs** **(39,700)**	**Probability of differential expression between the libraries**
***ACTB***	11	187	Prob > 0.999

***GAPDH***	31	225	Prob > 0.999

***HPRT1***	7	0	0.5 < Prob < 0.6

^1 ^*ACTB *and *GAPDH *are abundantly expressed housekeeping genes, while *HPRT1 *is expressed at low levels.

### Statistics of the sequencing results

**Figure 2:**
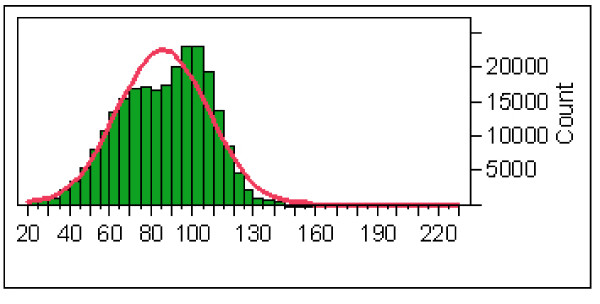
**Distribution and statistics of the cDNA reads**. Distribution and statistics of the non-redundant cDNA sequences for the initial nebulized 454 reads dataset (251,262 sequences). The independent variable (X axis) is the read length, the dependent variable is the sequence count corresponding to each length bin. The red line is an approximation to a normal distribution, with mean of 85.6 and an estimate dispersion of 22.1

We used the NCBI nrdb software to filter out technical redundancy from the sequence dataset. We obtained 251,262 non-redundant sequence reads, fitting approximately a normal distribution with a median length of 88 nt and the third quartile at 102 nt (Figure 2). After mapping the non-redundant reads to the genome (requiring a minimum coverage of 70%), we obtained a second dataset of 194,806 distinct sequences which excluded all reads with uncertain mapping. A threshold of 98% identity, 98% coverage and a single match on the genome was then used for comparisons with annotated transcripts, gene structures, highly conserved genomic regions, ENCODE regions, and ncRNAs, resulting in 132,113 reads (Table 2). This dataset was used for all the other statistics in this section and will be referred to as the 98.98.1 dataset. The 98.98.1 dataset has been deposited at the EMBL Nucleotide Sequence Database as EST sequences with the Accession Numbers FN045784 to FN177896.

**Table 2:** Primary classification of the 454 sequencing reads

**Set Description**	**Number of reads**
Total (unfiltered)	251,262

Mapping to the genome, 70% coverage, high stringency	194,806

**Subset with a single match on the genome at 98% identity and 98% coverage (98.98.1 dataset)^1^**	**132,113**

Subset with a single match on the genome and 100% coverage of the alignment^2^	114,427

Subset of 98.98.1 dataset matching with max 6 errors (mismacthes + indels) and 90% coverage on UCSC all_mrna and RefSeq – canonical transcripts dataset	59,632

Subset of 98.98.1 dataset matching inside an UCSC Known Gene (Intragenic dataset, intronic + exonic transcripts)	118,840

Matching with max 6 errors (mismatches + indels) and 90% coverage to the Human ORESTES EST dataset (764,587 sequences)	68,396

^1 ^Reference dataset

^2^87% of the reference dataset. This set was used for genomic classification (Table 3)

**Figure 3:**
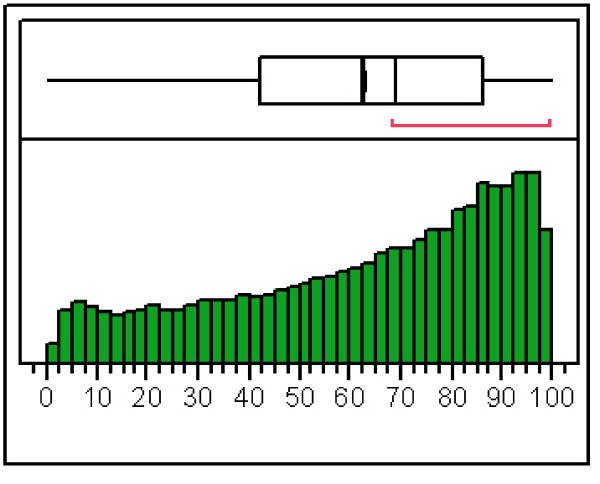
**Uniformity of sequence coverage across transcripts**. Sampling of an 'ideal' complete target transcripts by the 98.98.1 read dataset. 0 means that the 454 sequence identifies a point at the 5' of the target RefSeq transcript, while a value of 100 correspond to a sampling of the 3' end. The upper box is an Outlier Box plot, representing the interquartile range, the mean and the limits of the outliers. The red line represent the shortest area in which 50% of the data are represented. The total number of matches represented in this plot is 74,208.

The aim of cDNA nebulization was to maximize the sampling of sequence length. In order to evaluate the effective coverage of full-length transcripts obtained with our protocol, we counted all the high-quality Blat matches of the 98.98.1 sequence reads dataset mapping to the human RefSeq transcript database [, April 2008]. A total of 11,551 different RefSeq genes were identified with stringent parameters by 51,369 distinct sequence reads, corresponding to the 39% of the 98.98.1 dataset. Analysis of tag density across Refseq transcripts showed that cDNA nebulization generated reads randomly covering the whole length of a transcript, although with a clear oversampling of 3'untranslated regions (3'UTR) (Figure 3). This is not an unexpected finding, since RT-PCR followed by cDNA synthesis is necessarily biased toward the 3' end of the transcript, unless controlled partial hydrolysis of RNA is performed before retrotranscription [41].

One effective way of exploring molecular diversity by sequencing is through analysis of mRNA 3'UTRs, which are rich in single-feature polymorphisms that distinguish closely related transcripts. The specificity of 3'UTR sequences allows effective annotation of individual mRNAs without assembly of complete cDNAs and can be useful in transcriptome profiling by sequencing [42]. However, caution should be used in data interpretation, as there is some evidence that 3'UTRs may be separately expressed (Wilhelm, Soldà, Mercer, Dinger, Simons, Glazov, Koopman and Mattick, unpublished data). We used the non-redundant dataset of human 3'UTRs (39,758 sequences) from UTRdb, a curated database of 5' and 3' untranslated sequences of eukaryotic mRNAs [43], as a target for the 98.98.1 sequence reads dataset, requiring perfect identity and coverage of at least 90% to accept a match. From a total of 18,262 matches to the UTRdb we obtained 9,178 reads which could be univocally associated with a single RefSeq transcript (~50% of the matches). We conclude that the 454 reads mapping with high quality on a transcriptome have a high 'resolution power', or ability to distinguish between transcript variants

### Genomic classification of sequence reads

In order to characterize and annotate the breast cancer transcriptome, we mapped each read on the human genome and extracted all features associated with that target region. We then employed a hierarchical classification based on multiple criteria; the results are summarized in Table 3 and detailed in Additional file 2.

**Table 3:** Genomic classification of the 454 sequencing reads

**Sequence class**	**Number of reads**
**Intergenic Unspliced**	**6,298**

**Intergenic Spliced**	**402**

**Intragenic Unspliced – total**	**97,690**
*3 TERM*	*2,475*
*(Poli-A)*	*(989)*
*(INTERNAL)*	*(1,486)*
*5 TERM*	*2,807*
*(TSS)*	*(1,113)*
*(INTERNAL)*	*(1,694)*
*EXON*	*1,331*
*INTRAEXON*	*64,326*
*INTRON*	*26,751*

**Intragenic Spliced**	**10,037**

**Total**	**114,427**

Abbreviations: 3 TERM, read which extend the annotated 3'term of the target gene. Poli-A: read which extends at 3' the last exon. INTERNAL: read which extends at 3' any exon except the last. 5 TERM: read which extend the annotated 5' term of the target gene. TSS: read which extends at 5' the first exon. INTERNAL: read which extends at 5' any exon except the first. EXON: read mapping inside an exon with one or both ends coincident with exon boundaries. INTRAEXON: read mapping completely inside an exon of the target gene. INTRON: read mapping completely inside an intron.

The first clustering divided all the genome matching reads in two large datasets: 'spliced' and 'unspliced' reads (see Methods). The 'unspliced' dataset was split into intragenic or intergenic. Intragenic reads were then assigned to 4 different classes: exon, intron, extended 5' and extended 3'. The 'spliced' dataset was also classified by location within a gene. In order to detect potentially novel transcriptional features we excluded the entire unspliced-exon dataset from further analyses, as this will mostly contain well-known entities. We noticed that there are a significant number of matches in the intragenic non-exonic portion of genes, which we attribute to new exons, retained introns or intronic transcripts.

**Figure 4:**
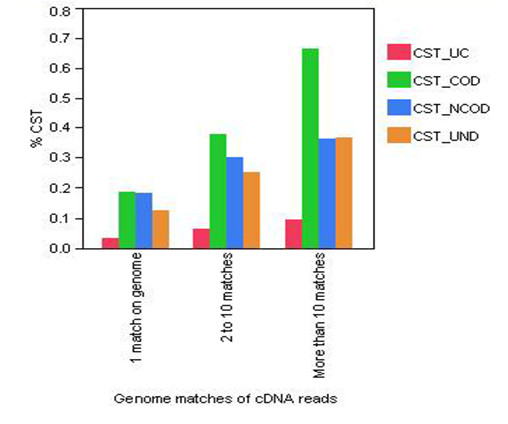
**Distribution of Conserved Sequence Tags (CST) in relation with cDNA genome mapping**. Plot of the percentage of the CST conserved segments overlapping cDNA reads for each of the following categories: matching only once in the genome; matching from 2 to 10 times; matching more than 10 times. CST_UC = CST with unknown coding potential; CST_COD = CST with coding potential; CST_NCOD = CST without coding potential; CST_UND = undetermined coding potential.

Genome-wide identification of coding and non-coding Conserved Sequence Tags (CST) in human and mouse genomes [34,35] provides a dataset of genome coordinates which can be correlated with our deep sequencing reads, especially those associated with putative novel transcripts. The distribution of the CSTs, divided in four categories (undefined; non-coding; coding; ultraconserved), is reported in Figure 4 and shows the normalized ratio between the numbers of the Conserved Sequence Tags in each category with the corresponding number of cDNA reads. The categories of the cDNA reads are a function of the number of high-quality matches for each of the reads: one single match on the genome; from 2 to 10 and more than 10. The sequences which match only once on the genome show an equal distribution between overlapping coding and non-coding CSTs, while sequences with multiple matches tend to overlap conserved regions with high coding potential.

### Identification and primary validation of potential cancer-associated transcriptional events

Detailed bioinformatic analyses were performed on our breast cancer library to identify fusion transcripts, aberrant or novel splicing isoforms, as well as known cancer-related splice variants (see Methods and Additional file 1). In total, we found 477 putative rearrangement events. It must be noted, however, that we expected the rate of false positives to be high, due to sequencing or PCR artifacts. A manually curated selected dataset, including only reads containing at least one end in proximity of a splice site, identified six putative translocation-mediated fusion events and two intragenic deletions; the relative sequences in FastA format are available from Additional File 3 and the genome mapping and annotation are in Additional file 4.

**Figure 5:**
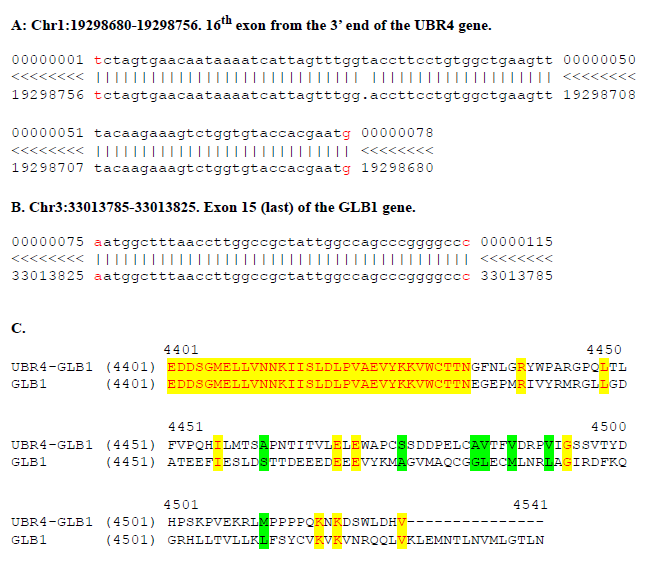
**Identification of a *UBR4/GBL1 *fusion transcript**. (A, B) Details at the nucleotide level of the fusion event. Splice junctions are indicated in red. (C) alignment of the divergent parts of the original *GLB1 *peptide and of the fusion *UBR4-GLB1*. Residues before 4,433 are identical. The yellow and green background identifies identical or conserved residues.

Fusion transcripts can be derived from either trans-splicing of separate pre-mRNA molecules [44] or from transcription of rearranged chromosomal regions in which sections of two separate chromosomes have been joined by translocation, deletion, or inversion [16,17]. A potentially interesting fusion event was detected from the sequence 107781_1044_1738 (115 nt long), which we renamed 4A, involving two genes located on different chromosomes: *UBR4 *(ubiquitin protein ligase E3 component n-recognin 4) on chromosome 1 and *GLB1 *(beta-galactosidase-like protein) on chromosome 3. *UBR4*, commonly known as p600 or retinoblastoma protein-associated factor 600, is a cellular target of the human papillomavirus type 16 E7 oncoprotein, contributing to anchorage-independent growth and cellular transformation. UBR4-E7 interaction strongly contributes to cellular transformation [45]. The *GLB1 *gene encodes beta-galactosidase-1 (EC 3.2.1.23), a lysosomal hydrolase that cleaves the terminal beta-galactose from ganglioside substrates and other glycoconjugates. The predicted fusion, verified by direct sequencing of the original cDNA library, links exon 16 of the gene *UBR4 *with the terminal exon (composed of coding and 3'UTR sequence) of the *GLB1 *gene. Our sequence is colinear with both transcripts and exon-exon junctions are clear in the hybrid sequence. The predicted final processed fusion cDNA *UBR4/GLB1 *would be 14,022-bp long and would produce a large protein of 4,526 residues, which is shorter than the original UBR4 protein (5,183 residues). The UBR4/GLB1 fusion protein is identical to UBR4 up to residue 4,433, and then diverges significantly (Figure 5). Fusion transcripts are common in haematological malignancies and they are also recognized as contributing to the pathogenesis of solid tumors [20]. Our sequencing approach thus allowed the reliable detection of one novel cancer-related gene fusion which we subsequently validated at the transcript level in the original sample, although we could not replicate clearly this finding in the other eight breast cancer RNAs examined (Additional file 5). Recent papers, which appeared while this work was under review, highlighted that a particular cancer cell line or tissue can harbour multiple gene fusions, many of which are likely not recurrent, in accordance with our results [16,17]. Further refinements to the current protocol, such as paired-end sequencing or coupling of long with short reads, will be more effective in the specific identification of fusion events. Indeed, a recent study using Paired-End diTags (PETs) in cancer cell genomes detected 70 new fusion transcripts [46].

**Figure 6:**
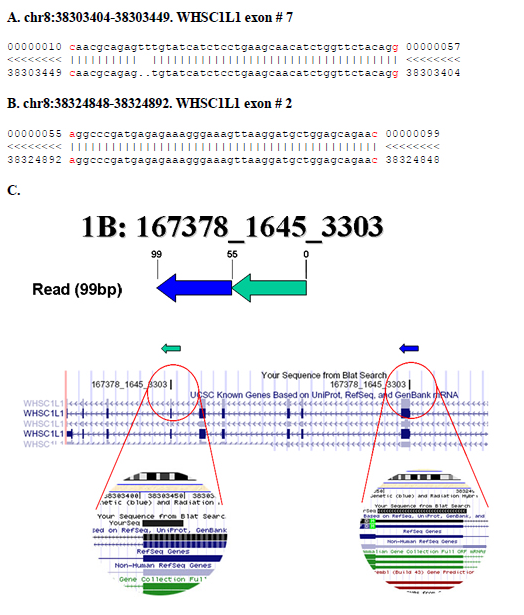
**Identification of a deletion within the *WHSC1L1 *gene**. (A, B) Alignment of the read 1B with the corresponding genome regions of *WHSC1L1*. Splice junction nucleotides are indicated in red. (C) Schematic representation of the putative intragenic deletion, (probably due to a looped transcript) in *WHSC1L1*, identified by the read 1B. The green and blue arrows represent the two halves of the fusion transcript which map on the opposite order to the genome.

An example of a transcriptional or genomic deletion event is provided by sequence read 1B (167378_1645_3303), located on chromosome 8. We interpret this sequence as a deletion, probably due to a loop that causes the inclusion of exons 2 and 7 of the *WHSC1L1 *gene in an inverted order in the mature transcript (Figure 6). This transcript was also confirmed by direct sequencing of the cDNA library and by RT-PCR in an additional breast cancer sample (Additional file 5). The *WHSC1L1 *gene is related to the Wolf-Hirschhorn syndrome candidate-1 gene and encodes a protein with PWWP (proline-tryptophan-tryptophan-proline) domains. Two alternatively spliced *WHSC1L1 *variants have been described. The long isoform contains a PHD-finger domain (an interleaved type of Zn finger chelating 2 Zn ions) and a SET domain (protein-protein interaction domain); however, the function of the protein has not been determined yet and hence the relevance to cancer aetiology of this deletion is uncertain.

The median length of the sequence reads in this sequencing run was around 85 nt, which is considerably longer than what can be achieved with other deep sequencing technologies, although much shorter than the length achievable with the latest generation of 454 sequencers (FLX and Titanium). This read length made it possible to identify interesting transcripts (ncRNAs; novel isoforms; fusions; deletions) without any sophisticated molecular '*a priori*' processing of the RNA, apart from library normalization. This approach may be particularly useful to researchers investigating the transcriptome of species with poor genome annotation.

**Figure 7:**
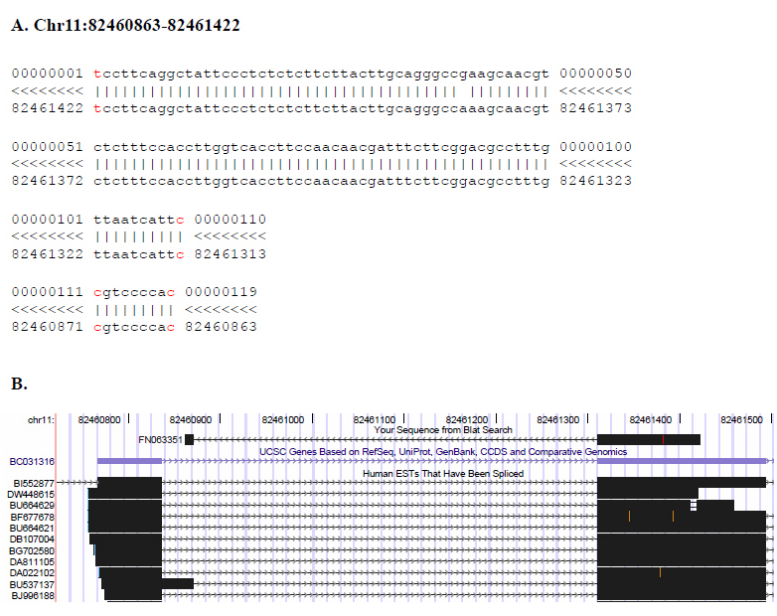
**Identification of a novel isoform of the **BC031316**gene**. (A) Alignment of the sequence read AI4 with the human genome. (B) UCSC human genome screenshot showing the novel isoform, with a longer first exon, of the annotated BC031316 gene, identified by the sequence read AI4 and further supported by many annotated spliced ESTs.

Taking advantage the length of 454 sequencing reads we investigated alternative splicing events, which are known to be significantly altered in breast tumors and can be used to identify normal and cancerous breast tumor tissue [47]. We identified a 102 nt read, 045624_1590_1179 (which we renamed 6B), which corresponds to a known isoform spanning the Variable, Light and Join segments of Unigene cluster 449585, Immunoglobulin lambda joining 3, on chromosome 22q11.1-q11.2. When mapped to the genome, this read aligns to three different exons, the second and the third being separated by ~511,000 bases (Additional file 4). This rare isoform was validated both by direct sequencing of the cDNA library and by RT-PCR of the original RNA, but was not detected in any other breast cancer sample tested (Additional file 5). We also identified splice isoforms that had not previously been reported. The 119 nt read 023612_1809_1670, renamed AI4, spans a splice junction of the gene BC031316 and is supported by three spliced ESTs but by no full-length mRNA. This isoform was confirmed by direct sequencing on the original cDNA library (Figure 7) and was detected by RT-PCR in multiple additional breast cancer samples (Additional file 5).

**Figure 8:**
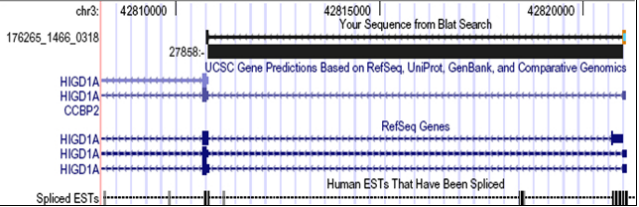
**Identification of a putative cancer-associated isoform**. UCSC genome screenshot showing the region where the 176265_1466_0318 read maps, clearly identifying the shorter protein isoform (1b) of *HIGD1A*, which is predicted to be significantly associated with the 'cancer' phenotype according to ASAPII EST and transcript analysis. The black block identified as 27858 is the ASAPII intron associated with the cancer-specific *HIGD1A *isoform 1b splice site.

Specific cancer-associated transcript isoforms have been predicted based on differential representation of ESTs between tumor and healthy tissues. When we queried the ASAPII database of alternative splicing with our deep sequencing reads we identified five putative cancer-associated isoforms, one of which has a plausible role in breast cancer biology. Isoform b of *HIGD1A *(HIG1 domain family, member 1A) gene (Figure 8) encodes a protein containing a domain likely to be involved in the hypoxia response. Growth and progression of breast cancers are accompanied by increased neovascularization (angiogenesis) and a variety of factors, including hypoxia, contribute to increased production of angiogenic factors [48]. The *HIGD1A *variant (isoform b) identified by deep sequencing contains a distinct 5'UTR and lacks a portion of the 5' coding region, compared to variant a. These differences cause translation initiation at a downstream AUG and a protein with a shorter N-terminus. The predicted association with cancer is strongly supported by the ASAPII LOD score for this transcript [49]. Our data suggest that specific isoforms of *HIGD1A *are expressed in breast cancer and may be associated with the disease state.

### Experimental validation of selected transcript variants identified with bioinformatic analysis

A subset of the above-described transcripts, including some potential cancer-associated variants, were selected for biological validation by RT-PCR on RNA derived from the same tissue sample that was used for deep sequencing, as well as from other eight lobular breast cancers. Amplification products from the original sample were assessed by Sanger sequencing. We obtained experimental confirmation of one deletion, one novel intergenic transcript, one novel intragenic transcript, nine previously unannotated isoforms – two associated with exon skipping and seven with novel splice site usage – and one known, but rare isoform. A gene fusion event which we detected by deep sequencing could be validated only in the original sample. These results, the primer sequences, the transcript labels and the PCR product sizes are described in Additional file 5.

### Detection of known and novel non-coding RNAs and of putative new transcriptional units

A number of approaches are currently available to discriminate protein-coding transcripts from ncRNAs [50]. The most reliable way of evaluating the percentage of sequences that could represent real matches with ncRNAs is a direct sequence comparison with stringent criteria and an appropriate error model for 454 sequencing reads [51] (see Methods). In order to examine the expression of ncRNAs in our breast cancer sample, we first screened known ncRNAs selected from two independent non-coding RNA databases: RNAdb and NONCODE [36,37]. In total, 98 sequences corresponding to known ncRNAs were detected in our library (Table 4 and Additional file 6). Interestingly, some of them such as *SRA1 *and *MALAT1*, have previously been associated with other tumor types and might also play a role in breast cancer pathogenesis. The steroid receptor RNA activator (*SRA*) is a unique modulator of steroid receptor transcriptional activity that functions as a regulatory RNA assembled in a ribonucleoprotein complex. Recent findings, however, have shown that the *SRA1 *locus can produce both protein-coding and non-coding transcripts which are involved in the regulation of estrogen and androgen receptor signaling pathways. Moreover, several reports have shown increased *SRA *expression in breast, uterus and ovarian cancers, and a possible direct involvement of *SRA *transcript in the mechanisms underlying breast tumorigenesis and tumor progression has been proposed [52].

**Table 4:** Annotation of the non-coding part of the transcriptome

**ncRNA class**	**Number of unique ncRNAs matching the breast cancer library^4^**
**Small RNAs:**	**24**
*piRNAs*	*23*
*scAluRNAs*	*1*

**Long regulatory RNAs^1^**:	**35**
*Host genes*^2^	*11*
*Imprinted transcripts*	*4*
*Antisense transcripts*	*9*
*Cancer associated transcripts*	*11*

**TUF**	**26**

**Expressed pseudogenes**	**11**

**Predicted conserved secondary structure^3^**	**2**

**Total**	**98**

^1 ^The same regulatory RNA may belong to more than one subclass.

^2 ^Both miRNA and snoRNA host transcripts are considered.

^3 ^According to RNASearch predictions [61].

^4 ^Known ncRNAs were retrieved from both RNAdb and NONCODE databases (see Methods)

Abbreviations: piRNA, Piwi-associated small RNAs; scAluRNA, small cytoplasmic Alu-repeat RNAs; TUF, transcripts of unknown function.

**Figure 9:**
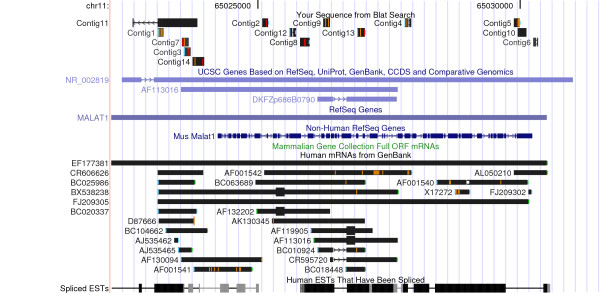
**Abundant representation of *MALAT1 *ncRNA in 454 sequences from the breast tumor sample**. UCSC human genome screenshot of the region containing the *MALAT1 *locus. 309 cDNA reads map with high confidence along MALAT-1, a ncRNA highly correlated with poor prognosis in several tumor types. These were assembled in 14 contigs, which are reported in the figure.

The most abundant ncRNA we detected was *MALAT1 *(metastasis associated lung adenocarcinoma transcript 1). *MALAT1 *is a conserved 8-kb ncRNA whose expression correlates with the risk of developing metastasis in non-small-cell lung cancer (NSCLC) patients. Recent studies have also reported the overexpression of *MALAT1 *in uterine endometrial stromal sarcoma and hepatocellular carcinoma [25,26]. We found 309 reads mapping along this regulatory ncRNAs, which, when assembled with the cap3 program [31], gave rise to 14 contigs (sequences available in Additional file 3) distributed along all the length of the ncRNA (Figure 9). Interestingly, only 6 of the 309 reads map to portion of *MALAT1 *which is cleaved in the nucleus and generates a cytoplasmic tRNA molecule [53]. This observation suggests that *MALAT1 *is abundantly expressed in our primary sample, in accordance with previous results [26], and prompted us to further investigate this finding.

We found that *MALAT1 *is abundantly expressed in all the publicly available annotated breast cancer samples retrieved from the CleanEx database [54]. Detailed analysis of an Affymetrix ER+ Tamoxifen-treated and untreated breast cancer data set [55] showed a relevant variation in *MALAT1 *transcript abundance, including a few outliers with very high expression of this ncRNA. Further analysis of cDNA microarrays, probed with total polyA+ RNA from ER+ lobular and ductal breast cancers treated with Tamoxifen, showed the same expression patterns [56]. This reinforces our finding that high *MALAT1 *expression may be episodically associated with single breast tumors and that the sensitivity of our deep sequencing approach facilitated the detection of this ncRNA in our sample. We also noticed that the Coefficient of Variation of gene expression values was higher in Tamoxifen-treated versus Tamoxifen-untreated breast cancer samples (84% versus 43% for the Affymetrix array experiments dataset) (Additional file 5).

Surprisingly, we found 23 reads corresponding to PIWI-interacting RNAs (piRNAs), which are thought to be selectively expressed in male and female gonads and are important for the control of transposable elements during germline development [7]. However, piRNA expression in breast cancer is not totally unexpected. A mechanism of piRNA biogenesis that is not confined to the germline has recently been described [57].

**Figure 10:**
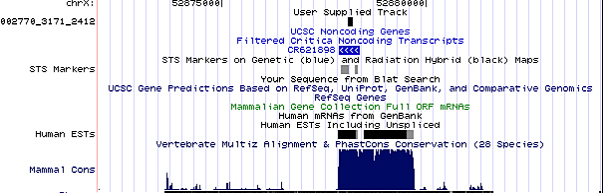
**Identification of a novel conserved noncoding transcript**. UCSC human genome 8 kb screenshot of a "gene desert region" (no known gene in a 50 kb boundary) on the X chromosome tagged as transcribed by the sequencing read 002770_3171_2414. The read overlaps a CRITICA-predicted putative noncoding transcript (CR621898) and points to a new, highly conserved transcriptional island, according to a vertebrate 28 multi-species alignment and PhastCons conservation score.

Sequences that mapped to 'gene deserts', which lie at least 2 kb from the boundaries of any known transcript, and which resulted highly conserved according to UCSC PhastCons17, were manually examined for overlap with ESTs, transcript predictions and non-coding RNAs annotations derived from CRITICA [2,8,58]. Eleven per cent (684 of 5,950) of the reads that mapped to 'gene deserts' actually overlap CRITICA-predicted non-coding transcripts or are supported by EST data. These reads likely represent non-protein-coding genes (Additional file 6 and Figure 10), or exons at a significant distance (greater than 5 kb) from a gene, belonging to exceptionally extended 3'UTRs. In agreement with our data, evidence of extended 3'UTR has been recently reported in a deep-sequencing screen of the mouse transcriptome [41]. We also compared the reads mapping at least 5 kb from any known transcript (1,069 sequences) with a collection of sequences that are highly conserved between human and mouse and which are classified in order of coding potential (CSTs) [35]. We were able to identify 314 reads overlapping one or more CSTs with coding potential, and a further 351 reads overlapping non-coding CSTs. These results suggest that our deep sequencing and bioinformatics protocols are capable of detecting rare and novel transcripts outside known gene structures (Additional file 7).

The ENCODE annotation of transcriptionally active regions [38] (Transcripts of Unknown Function: TUFs) covers only 1% of the genome. However, we found 135 reads that overlap with 60 distinct ENCODE TUFs. We identified individual TUFs with both single and multiple reads, confirming our protocol's efficacy in enriching for non-canonical transcripts (Additional file 8).

### Functional annotation of the coding part of the sequenced transcriptome

Functional annotation of transcripts has become an important aspect of microarray studies, and many tools are now available to assess gene expression biases [59]. Using the functional annotation strategies that are usually applied to microarray experiments functional annotation, we examined the genes identified by deep sequencing. 454 reads mapped to 6.067 RefSeq transcripts (Additional file 9) with counts per transcript ranging from 82 to 1, with median of 2. *AKAP9*, which interacts with multiple signal transduction pathways, had the highest number of counts, followed closely by the ncRNAs *MALAT1 *and *XIST*. Among transcripts with very few counts we identified a number of annotated pseudogenes.

**Figure 11:**
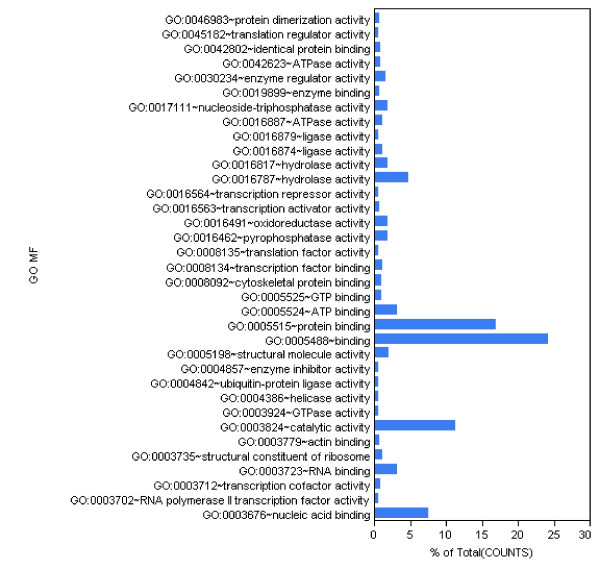
**Mapping the transcriptome to GO Molecular Function**. GO Molecular Function mappings to the genes identified by the cDNA reads correlated with RefSeq transcriptome.

We applied the DAVID on-line analytical tool [60] to identify enrichment of specific Gene Ontology (GO) terms among the genes which had at least two cDNA read counts (3,589 genes). The most enriched categories are related with protein and nucleic acid binding and with catalysis, as expected in an actively proliferating tissue (Figure 11).

## Conclusion

Quantitative transcriptional analysis of all the genes expressed by breast tumors has provided the first steps towards defining a molecular signature for the disease, and might ultimately make conventional diagnostic techniques obsolete. The qualitative analysis of the breast cancer transcriptome – such as the one obtained by massive cDNA sequencing and presented here – should instead contribute different and complementary information: the identification of novel possible pathogenic determinants (gene fusions and genome deletions) or biomarkers (aberrant or novel transcripts and isoforms, intronic and extragenic ncRNAs, expressed pseudogenes).

We demonstrated in this work that 454 deep sequencing of a normalized cDNA library, coupled with detailed biology-oriented bioinformatic analyses, has the potential to identify transcripts that may further our understanding of the breast cancer transcriptome, even starting from a relatively small number of sequences. In our primary breast cancer cDNA library and in a number of additional samples with a matching histotype, we have identified and validated several unusual transcriptional events that could be suitable for subsequent functional studies: gene fusions, gene deletions, novel or cancer-associated isoforms and putative novel ncRNAs.

We have also identified from our sequences a very high expression of the cancer-associated *MALAT1 *ncRNA and we replicated this observation in two different gene expression profiling experiments of well-annotated ER+ breast cancer patient cohorts, finding also an high variance between Tamoxifen treated and untreated patient samples. Although further technical refinements, such as controlled hydrolysis of RNA samples before cDNA synthesis and paired-end or di-tags sequencing, can increase significantly the number and diversity of sequences which can be annotated, our protocol has proved to be very effective in detecting rare or novel transcriptional events. Based on the results presented here, we are confident that further deep sequencing experiments and a similar bioinformatic analysis strategy will yield an even more comprehensive and detailed picture of the breast cancer transcriptome.

## Authors' contributions

AG planned and coordinated all the bioinformatic analyses, performed the statistical analysis and functional characterization part and wrote the manuscript. MI contributed the genome mapping and read classification. PP and IZ prepared the normalized cDNA library and performed the biological validation. MAA performed experimental validations on the ncRNAs part. NK performed the bioinformatic search for gene fusions, deletions and cancer-associated isoforms. LJC and RJT performed the analyses on novel ncRNAs and contributed to the manuscript. GS performed the analyses on known ncRNAs, and contributed to the manuscript. ER performed the deep sequencing. MC contributed the bioinformatic analysis of breast cancer cDNA array data. RJB contributed computational and bioinformatic support to this project. FM and GP contributed the CST bioinformatic analysis. GB contributed the biological sample and pathological characterization. LRB and AA conceived this research project and established the deep sequencing laboratory. CL contributed to the cancer isoforms detection analysis. JSM contributed to the manuscript and coordinated the ncRNA analysis part. GdB planned and coordinated the deep sequencing work and contributed to the manuscript.

## Supplementary Material

Additional file 1 **Supplementary methods**. document detailing the methods for the assessment of library normalization; mapping to transcriptome and genome; identification of cancer-specific splice sites and fusion/deletion transcripts; analysis of non-protein coding transcripts. Click here for file

Additional file 2 **Genomic classification of cDNA reads**. Excel document containing the identifiers and annotations of all the mapped cDNA reads classified as described in Table 3. Click here for file

Additional file 3 **FASTA sequences of cDNA reads corresponding to fusions, deletions, new transcripts and *MALATI***. WinRAR zip archive containing three multifasta text files: (1) Fusion_Deletions: cDNA reads corresponding to fusions and deletions described in Additional file 4; (2) New_Transcripts: cDNA reads corresponding to the extragenic new transcripts supported by ESTs described in Additional file 6; (3) Malat.fasta: contigs generated from the assembly of sequence reads corresponding to the *MALATI *ncRNA. Click here for file

Additional file 4 **Annotation of cDNA reads corresponding to fusions, deletions and a rare isoform**. Word document containing sequence analysis details of the all fusions and deletions (validated and non validated) predicted from the analysis of cDNA reads, plus an example of a (validated) rare isoform. Click here for file

Additional file 5 **Biological validation of selected interesting transcripts**. Word document containing the description of the RT-PCR validations for potential cancer-related transcripts identified in this study; the reanalysis of two Affymetrix and cDNA array breast cancer patients datasets for the investigation of the *MALAT1 *ncRNA expression pattern. Click here for file

Additional file 6 **Analysis of cDNA reads corresponding to known and novel ncRNAs**. Excel document containing the list, genomic coordinates and annotation of reads corresponding to known ncRNAs supported by ESTs; 'desert' and 'intronic' reads overlapping with CRITICA non-coding RNAs; conserved extragenic cDNA reads corresponding to novel or extended transcripts supported by ESTs. Click here for file

Additional file 7 **Extragenic cDNA reads overlapping with CSTs**. Excel document containing the list and genomic coordinates of extragenic cDNA reads overlapping CSTs with or without coding potential. Click here for file

Additional file 8 **cDNA reads overlapping with ENCODE transcripts**. Excel document containing the identifiers and genomic coordinates of the cDNA reads overlapping the ENCODE transcripts subset supported by microarray evidence (meta analysis). Click here for file

Additional file 9 **Annotation and count of cDNA reads corresponding to known genes**. Excel document containing the HUGO gene identifiers corresponding to the mapped cDNA reads and the relative count. Click here for file
