# Supplementary material for: A transcriptional sketch of a primary human breast cancer by 454 deep sequencing
Source: BMC Genomics. 2009 Apr 20;10:163. doi: 10.1186/1471-2164-10-163 (PMC2678161; doi:10.1186/1471-2164-10-163)
Supplement: Additional file 1 — Supplementary methods. document detailing the methods for the assessment of library normalization; mapping to transcriptome and genome; identification of cancer-specific splice sites and fusion/deletion transcripts; analysis of non-protein coding transcripts. [file 1471-2164-10-163-S1.doc]

**Additional file 1. Supplementary Methods**

1. **cDNA library normalization and assessment of library normalization before and after sequencing**

Briefly, the normalization process uses second-order reaction kinetics of re-association of denatured cDNA, so that relative transcript concentrations within the remaining single-stranded cDNA fraction were equalized to a considerable extent. The equalized portion was isolated by treatment with kamchatka crab double-strand nuclease (DSN), a thermostable enzyme that specifically hydrolyzes nucleic acid duplexes (Zhulidov *et al.*, 2004), followed by an amplification of the single-stranded uncleaved cDNA. The developed protocol included a thermal denaturation followed by re-association of the cDNA performed at 70°C for 5 hours. The enzymatic digestion was performed at 70°C (taking advantage of the stability of DSN) to prevent random annealing. Final amplification of the normalized single-stranded cDNA was carried out using Long Distance PCR.

1. Three reference genes were selected to assess library normalization before and after sequencing. *ACTB* (Beta-actin) and *GAPDH* (Glyceraldeide-3-phosphate dehydrogenase) are expressed at relatively high levels in many different tissues, while *HPRT* (Hypoxanthine phosphoribosyltransferase 1) is expressed at low levels (Zhulidov 2004). PCR amplifications of the three target on cDNA before and after normalization were performed using specific primers, reported in Table I, and standard thermal cycling conditions.
2. To assess whether the library normalization was also reflected in the 454 sequence output, we counted the reads that could be unequivocally associated with *ACTB*, *GAPDH* and *HPRT* among the sequences which mapped in a single location with the human genome at 98% coverage and 98% identity (132.113 sequences). We compared these matches with the EST counts corresponding to the same transcripts identified in a public domain EST library collection with the same tissue and pathology of our experimental sample. We finally applied a well-established statistical test for assessing significant differences in digital gene expression profiles (Audic 1997) to evaluate the probability for any significant difference in the extrapolated expression of these genes.
3. The target EST library (total of 39,700 sequences) used for the comparison was the merge of: NCI_CGAP_Br3 (female breast tumor tissue, non-normalized, Unigene Lib 986), NCI_CGAP_Br12 (female breast invasive carcinoma, non-normalized, Unigene Lib. 1443), NCI_CGAP_Br22 (invasive breast ductal carcinoma, 3 pooled samples, non-normalized, Unigene Lib. 7210), NIH_MGC_87 (female breast tumor, non-normalized, Unigene Lib. 8582) and NIH_MGC_107 (female breast tumor, non-normalized, Unigene Lib. 10554).

| Gene | Forward Primer | Reverse Primer |
| --- | --- | --- |
| *Actb* | ACTCTTCCAGCCTTCCTTCC | TGATCTCCTTCTGATCCTG |
| *GAPDH* | CATCAGCAATGCCTCCTGCA | GAACATCATCCCTGCCTCTA |
| *HPRT1* | CGTGGGGTCCTTTTCACCAGCAAG | AATTATGGACAGGACTGAACGTC |

1. **Mapping sequencing reads to the transcriptome and genome**
2. All the database searches against known transcripts (such as ESTs) were performed using the NCBI BlastN program with G = 0 and W = 4 options, since the most frequent error-type in 454 sequencing is the addition of nucleotides in polyC or polyG tracts, related to indels in homopolymeric tracts. Post-processing of the Blast results was performed by requiring a coverage of the query read on the subject of 95% and a maximum of three errors (gaps + indels).

Non-redundant sequence reads were compared with the human genome using the Blat program (Kent 2002) with the –tileSize=8 option, and the results were filtered for maximum six errors (gaps + mismatches) and a minimum coverage of 90% (alignment length/query length). All human full-length transcripts annotated in UCSC database (all_mrna Table, all Human mRNAs from GenBank, human genome release hg18, March 2006) (Kuhn 2007) were used as reference set for the classification. We define a read ‘spliced’ if it maps on a chromosome with a coverage >= 95% and presents a gap >= 50 nt. We classify a read ‘intragenic’ if it maps at least partially within a known gene (either in an exonic or intronic region), otherwise it will be classified as ‘intergenic’. Additional criteria were used to define an “exon-oriented” classification. For instance, ”exonic” reads map completely within a know exon, while “intronic” reads map completely outside an exon, at a distance of more than 5 nt from the closest flanking exon. Finally, reads mapping across an exon junction were defined as ‘extended 5’or ‘extended 3’’.

1. A collection of Conserved Sequence Tags (CSTs) (Boccia 2005, Mignone 2008), obtained by a full-genome comparison of human and mouse genomes, was compared to the genome mappings of the cDNA reads, excluding reads located within known exon, to evaluate both conservation and coding propensity.

All the statistics were evaluated and plotted using the JMP7 software from SAS [http://www.jmp.com]

1. **Identification of cancer-specific splice sites and fusion/deletion transcripts**
2. For the detection of gene fusions and deletions, the minimum length of the reads used for the analysis was 50 bp, with at least 90% identity and a maximum of 3-bp insertions. First, chromosomal rearrangement were identified from read alignments consisting of two segments each mapping to a different location in the genome. Therefore, low coverage segmented alignments were diagnostic of a rearrangement. For a given segmental alignment, each non-overlapping segment was required to be at least 25-bp long and with the break point occurring within 10 bp of the nearest known splice site, separately for both front and rear segments. A small overlap between two segments was allowed due to the possible similarity between 5’ and 3’ fusion exons. Second, to identify reliable fusion transcripts by chromosomal translocation, we required that the breakpoint should be within the intronic region and the two gene directions at each breakpoint should be the same, on the rationale that as transcription occurs along those rearranged chromosomes, the putative fusion transcript should be joined exactly at the exonic boundaries of both genes. By comparing the gene direction of the breakpoints of putative fusion transcripts with known exon boundaries, we identified putative translocation-mediated interchromosomal fusion transcripts.
3. **Analysis of non-protein coding transcripts**
4. The breast cancer cDNA library was blasted against the UCSC Known Genes (human genome release hg18, 260.731 sequences) FastA sequences filtering for a maximum of 6 errors (mismatches + gaps) and 90% coverage, without any constraints on the identity percentage. All the reads that found a significant match according to these stringent criteria were classified as ‘exonic’. The genome coordinates of the reads were compared with the ‘txStart’ and ‘txEnd’ coordinates of the same class of transcripts from UCSC. Intragenic reads that did not belong to the first set (matching with exons) were considered as ‘intronic’. Reads mapping between two transcripts at a distance lower than 5 kb were classified as 'extragenic', while reads that could not be correlated with any transcript in the same window were tagged as ‘desert’. The read coordinates were then correlated with the UCSC PhastCons17 conservation score and four different datasets were generated: intronic, extragenic, desert conserved and desert nonconserved. All these datasets were crossed with ncRNA predictions as implemented by the CRITICA algorithm (Mercer et al. 2008).
5. As reference datasets for known ncRNAs we used a subset of RNAdb, a database specific for mammalian non-coding transcript, as well as NONCODE, an integrated knowledge database dedicated to non-coding RNAs. The human snoRNA and scaRNA, the known ncRNAs curated from literature, the ncRNA Search, and the Human Invitational datasets were downloaded [http://research.imb.uq.edu.au/rnadb/FastaDownloads/default.aspx] from the RNAdb website and compared against our reads. Detected matches were then analyzed and annotated manually in order to eliminate false positives and redundancies among the datasets.
