# Supplementary material for: A transcriptional sketch of a primary human breast cancer by 454 deep sequencing
Source: BMC Genomics. 2009 Apr 20;10:163. doi: 10.1186/1471-2164-10-163 (PMC2678161; doi:10.1186/1471-2164-10-163)
Supplement: Additional file 4 — Annotation of cDNA reads corresponding to fusions, deletions and a rare isoform. Word document containing sequence analysis details of the all fusions and deletions (validated and non validated) predicted from the analysis of cDNA reads, plus an example of a (validated) rare isoform. [file 1471-2164-10-163-S4.doc]

**Additional file 4: annotation of reads corresponding to
putative gene fusions, deletions and rare isoform**

**Putative fusion 1: validated by RT-PCR and Sanger sequencing: 4A**

**Alignment of 107781_1044_1738 and chr1:19298680-19298756**

**107781_1044_1738 115 0 78 chr1 247249719 19298679 19298756**

gtgaattatc aaccattgcc gtaaggtgcc ctttgctctt ggattttact 19298807

tgtcttacaa ttatttggaa caacagcttc acctgctttt ctgttcagct 19298757

TCTAGTGAAC AATAAAATCA TTAGTTTGGA CCTTCCTGTG GCTGAAGTTT 19298707

ACAAGAAAGT CTGGTGTACC ACGAATGagg tatgtgcctg ctttctcgtt 19298657

tggaggtgtg gtttcacttt tctgacttat gcagacttgt accagcagga 19298607

gccattgttc atcatcactt acctggc

00000001 tctagtgaacaataaaatcattagtttggtaccttcctgtggctgaagtt 00000050

<<<<<<<< ||||||||||||||||||||||||||||| |||||||||||||||||||| <<<<<<<<

19298756 tctagtgaacaataaaatcattagtttgg.accttcctgtggctgaagtt 19298708

00000051 tacaagaaagtctggtgtaccacgaatg 00000078

<<<<<<<< |||||||||||||||||||||||||||| <<<<<<<<

19298707 tacaagaaagtctggtgtaccacgaatg 19298680

**Alignment of 107781_1044_1738 and chr3:33013785-33013825**

**107781_1044_1738 115 74 115 chr3 199501827 33013784 33013825**

tggcctgccc tacatcaggg cccccacctt cctcagggct ttcggtgcat 33013876

ctggggttga tggttctctg tctctctctc tccagggcca ggtctggatt 33013826

AATGGCTTTA ACCTTGGCCG CTATTGGCCA GCCCGGGGCC Ctcagttgac 33013776

cttgtttgtg ccccagcaca tcctgatgac ctcggcccca aacaccatca 33013726

ccgtgctgga actggagtgg gcaccctgca gcagtgatga t

00000075 aatggctttaaccttggccgctattggccagcccggggccc 00000115

<<<<<<<< ||||||||||||||||||||||||||||||||||||||||| <<<<<<<<

33013825 aatggctttaaccttggccgctattggccagcccggggccc 33013785

**Putative Fusion 2**

**Alignment of 232305_2101_0218 and chr16:45092252-45092303**

**232305_2101_0218 99 0 49 chr16 88827254 45092251 45092303**

tcgcaatttt aatgtcctca agaaaattct tttccttttg ctggttccaa 45092201

tttttcagtg catctatttc cagtcttagc cttgcaattt cttcctgcaa 45092251

CATGCAAtTT TTATGCAACA GATCTTTTtc tTTCTTATGC CCATGAGAAA 45092301

TCtaaataaa caaaggaaac ttttagctaa cattcaatac aatgacattt 45092351

cattactttc tctgaaatta aagaatatcc tgcacattta tacaatgaaa 45092401

gc

00000001 catgcaa.ttttatgcaacagatctttt 00000027

>>>>>>>> ||||||| |||||||||||||||||||| >>>>>>>>

45092252 catgcaatttttatgcaacagatctttt 45092279

00000029 ttcttatgcccatgagaaatc 00000049

>>>>>>>> ||||||||||||||||||||| >>>>>>>>

45092283 ttcttatgcccatgagaaatc 45092303

**Alignment of 232305_2101_0218 and chr11:66162753-66162799**

**232305_2101_0218 99 48 95 chr11 134452384 66162752 66162799**

gtggagactg cggacctacc aggacacccc cgaagcccaa gggagtgaga 66162850

gaagatttaa cacagtacaa gaaaggcagt ggaaaggctc ggagtctcac 66162800

CCGCACAGAA ACCAGCAGGG CCTCCTCCTC GCGGCGCGGA CGCTTCTgac 66162750

aaaacgctaa aatggcggcg gccgcagcag tgctctgagt agaaggggga 66162700

ggtgagccta caaccggatt ggccactcaa gaaagtaaaa ggccggc

00000049 ccgcacagaaaccagcagggcctcctcctcgcggcgcggacgcttct 00000095

<<<<<<<< ||||||||||||||||||||||||||||||||||||||||||||||| <<<<<<<<

66162799 ccgcacagaaaccagcagggcctcctcctcgcggcgcggacgcttct 66162753

**Putative Fusion 3**

**Alignment of 226083_1454_2579 and chr8:49118144-49118201**

**226083_1454_2579 95 35 92 chr8 146274826 49118143 49118201**

gaaataattt ataaaggtta atttggaaaa agctgaattt aaatacgtgt 49118093

agctcttata attatgagct ttttacattt acactgacgt tcttttgtat 49118143

AGGAGTTAAA GTTCCTCGTA ATTTTCGCTT GTTGGAAGAA CTTGAAGAAG 49118193

GaCAAAAAgg agtaggcgac ggtacagtta gctggggcct tgaagatgat 49118243

gaagatatga cacttacaag gtggacaggc atgattattg ggccaccaag 49118293

ggtcagtg

00000036 aggagttaaagttcctcgtaattttcgcttgttggaagaacttgaagaag 00000085

>>>>>>>> |||||||||||||||||||||||||||||||||||||||||||||||||| >>>>>>>>

49118144 aggagttaaagttcctcgtaattttcgcttgttggaagaacttgaagaag 49118193

00000086 g.caaaaa 00000092

>>>>>>>> | |||||| >>>>>>>>

49118194 gacaaaaa 49118201

**Alignment of 226083_1454_2579 and chr1:200193283-200193318**

**226083_1454_2579 95 0 37 chr1 247249719 200193282**

gatattactg gtggtcccat cagtggctta gaaatgtact tctaatatat 200193232

tttttgttgc ttcctttttt ttgttatagg gagtaaacca cagactacga 200193282

GGGAGTTTGA CAGCTATTAA AACCAGGGCT CCACAGttag gaggtaagca 200193332

gaattttcat tttaactgaa ctattttttt ctgttccttt gaagatggag 200193382

ctatttttat ttttagatta caacaattag tgattt

000000001 gggagtttgacagctattaaaaccagggcttccacag 000000037

>>>>>>>>> |||||||||||||||||||||||||||||| |||||| >>>>>>>>>

200193283 gggagtttgacagctattaaaaccagggct.ccacag 200193318

**Putative Fusion 4**

**Alignment of 263771_1953_3434 and chr15:38115911-38115989**

**263771_1953_3434 117 0 79 chr15 100338915 38115910 38115989**

gatttttggc taccctaaat ccattatgca gatagggctg gtgttctgcc 38116040

agtttgcaca tcttcccact aaggtatgct ctgttgtatc tttcaggctt 38115990

ATTCAAACCT CCTTAGAGCT AACATGGATG GGcTGAAGAA GAGAGACAAA 38115940

AAGAACAAAA CTAAGAAGAC CAAAGCAGCa gcagcagcag cagcagcagc 38115890

acctgccgca gcagcaacag caccaacaac agcagcaaca acagcagcaa 38115840

cagcagcaca gtaaagggca tacatttcc

00000001 attcaaacctccttagagctaacatggatgggttgaagaagagagacaaa 00000050

<<<<<<<< |||||||||||||||||||||||||||||||| ||||||||||||||||| <<<<<<<<

38115989 attcaaacctccttagagctaacatggatgggctgaagaagagagacaaa 38115940

00000051 aagaacaaaactaagaagaccaaagcagc 00000079

<<<<<<<< ||||||||||||||||||||||||||||| <<<<<<<<

38115939 aagaacaaaactaagaagaccaaagcagc 38115911

**Alignment of 263771_1953_3434 and chr1:241534637-241534667**

**263771_1953_3434 117 86 117 chr1 247249719 241534636 241534667**

tgggtaaaaa gaaaaaaatt gagtttaata ttaaaaatta aagtttactt 241534586

ataaaataca ggacatagag aaaaatgtac ttctattttt cttttctata 241534636

GGAGAAGCTA AAACTTACTT ATGAGGAAAA Gtgtgaaatt gaggaatccc 241534686

aattgaagtt tttgaggtaa agtgaaatcg tccatttata gtcataccaa 241534736

aagcataatg atcttaaaat atatttgaat g

000000087 ggagaagctaaaacttacttatgaggaaaag 000000117

>>>>>>>>> ||||||||||||||||||||||||||||||| >>>>>>>>>

241534637 ggagaagctaaaacttacttatgaggaaaag 241534667

**Putative Fusion 5**

**Alignment of 192202_0091_1688 and chr5:167976229-167976281**

**192202_0091_1688 97 0 54 chr5 180857866 167976228 167976281**

aatcacatgc tccttgttct gcagcttggt gcggatggac atgataactt 167976332

ggccagtgtg aaccctggcc aaagtgccct ggggctttcc aaaggcacct 167976282

CGCATGCCTG TTTGGAGCCT GTCAGCCCCA GCACAGGACA ACATCTTGTT 167976232

GATgcggatg acgtggaagg agtggagccg cacccggata tggaagccat 167976182

ctttgccaca actttttacc atgtacttat tggcacaaat tcgggcagcc 167976132

tcc

000000001 cgcatgcctgtttggagcctgtcagcccccagcacaggacaacatcttgt 000000050

<<<<<<<<< ||||||||||||||||||||||||| |||||||||||||||||||||||| <<<<<<<<<

167976281 cgcatgcctgtttggagcctgtcag.ccccagcacaggacaacatcttgt 167976233

000000051 tgat 000000054

<<<<<<<<< |||| <<<<<<<<<

167976232 tgat 167976229

**Alignment of 192202_0091_1688 and chr2:215934216-215934258**

**192202_0091_1688 97 54 97 chr2 242951149 215934215 215934258**

accctgggag tttcctgagg gttttctcat aaatgagggc tgcacattgc 215934309

ctgttctgct tcgaagtatt caataccgct cagtatttta aatgaagtga 215934259

TTCTAAGATT TGGTTTGGGa TCAATAGGAA AGCATATGCA GCCaaccaag 215934209

atgcaaatgt tttgaaatga tatgaccaaa attttaagta ggaaagtcac 215934159

ccaaacactt ctgctttcac ttaagtgtct ggcccgcaat act

000000055 ttctaagatttggtttggggtcaataggaaagcatatgcagcc 000000097

<<<<<<<<< ||||||||||||||||||| ||||||||||||||||||||||| <<<<<<<<<

215934258 ttctaagatttggtttgggatcaataggaaagcatatgcagcc 215934216

**Putative Fusion 6**

**Alignment of 182139_1435_3797 and chr17:4648257-4648308**

**182139_1435_3797 113 0 53 chr17 78774742 4648256 4648308**

accattggca gtgaattgca gacactcttc cttggtcatg ccttcccggt 4648359

aggtagcatc aacatagcca tagatgtagg agctcccgga gcctccaatg 4648309

GCAAAGGACT GCCTTACCAT CATACCCCCC ATAGGCACTG AGTACACCTG 4648259

CCagatagaa ggagaggagt caccagtcgt atcctccctc caacacacgt 4648209

acattcacct gtccattctg ctattggtct gaggcacgtt aatagtaaga 4648159

at

0000001 gcaaagggactgccttaccatcataccccccataggcactgagtacacct 0000050

<<<<<<< ||||| |||||||||||||||||||||||||||||||||||||||||||| <<<<<<<

4648308 gcaaa.ggactgccttaccatcataccccccataggcactgagtacacct 4648260

0000051 gcc 0000053

<<<<<<< ||| <<<<<<<

4648259 gcc 4648257

**Alignment of 182139_1435_3797 and chr14:69303670-69303721**

**82139_1435_3797 113 61 113 chr14 106368585 69303669 69303721**

tcctccgcaa agagagcagc cccggccgct cacgatctga gcggccatga 69303772

agaagcctcg atccgagaag gcttgccctc aaagtgagcc actcacctac 69303722

CGGACGGGGT CTTTGGCGGC TGAGACCCAG ACAGGTCCGT AGTCTGCGAC 69303672

GGagtcgtcg acctccacgc agaccaggac ttaactcgtc tacgccactc 69303622

cacaactgac gcacttagca gctctggcgc tccacaaaat ggagggcgcg 69303572

tc

00000062 cggacggggtctttggcggctgagacccagacaggtccgtagtctgcgac 00000111

<<<<<<<< |||||||||||||||||||||||||||||||||||||||||||||||||| <<<<<<<<

69303721 cggacggggtctttggcggctgagacccagacaggtccgtagtctgcgac 69303672

00000112 gg 00000113

<<<<<<<< || <<<<<<<<

69303671 gg 69303670

**Putative deletion 1: validated by RT-PCR and Sanger sequencing: 1B**

**Alignment of 167378_1645_3303 and chr8:38303404-38303439**

**167378_1645_3303 79 1 37 chr8 146274826 38303403 38303439**

ttttaaaggg aattggtaac aaaacagaaa taagtgtcag ggggcaagac 38303490

aggcttataa tttctacacc aaaccagaga aatgaaaagc caacgcagag 38303440

TGTATCATCT CCTGAAGCAA CATCTGGTTC TACAGGttgg tatgaatgaa 38303390

aagtatatat aggtccattt tgagaaatcc ttgttctgtt ctgctataat 38303340

gtggtagctg tgtttctaag aaactcttag ttatca

00000002 tgtatcatctcctgaagcaacatctggttctacagg 00000037

<<<<<<<< |||||||||||||||||||||||||||||||||||| <<<<<<<<

38303439 tgtatcatctcctgaagcaacatctggttctacagg 38303404

**Alignment of 167378_1645_3303 and chr8:38324848-38324892**

**167378_1645_3303 79 34 79 chr8 146274826 38324847 38324892**

aaaattgagt tcaaaacatt ttagtttaat cctttttcag ttgattagcc 38324943

tctagcctat gagaatagta acaccttcta atcccctctt ttataatttt 38324893

AGGCCCGATG AGAGAAAGGG AAAGTTAAGG ATGCTGGAGC AGAACaatgg 38324843

atttctcttt ctctttcatg caagggatca tgggaaacac aattcagcaa 38324793

ccacctcaac tcattgactc cgccaacatc cgtcaggagg atgcc

00000035 aggcccgatgagagaaagggaaagttaaggatgctggagcagaac 00000079

<<<<<<<< ||||||||||||||||||||||||||||||||||||||||||||| <<<<<<<<

38324892 aggcccgatgagagaaagggaaagttaaggatgctggagcagaac 38324848

**Alignment of 117737_0258_2524 and chr8:38303404-38303438**

**117737_0258_2524 65 0 35 chr8 146274826 38303403 38303438**

tttaaaggga attggtaaca aaacagaaat aagtgtcagg gggcaagaca 38303489

ggcttataat ttctacacca aaccagagaa atgaaaagcc aacgcagagt 38303439

GTATCATCTC CTGAAGCAAC ATCTGGTTCT ACAGGttggt atgaatgaaa 38303389

agtatatata ggtccatttt gagaaatcct tgttctgttc tgctataatg 38303339

tggtagctgt gtttctaaga aactcttagt tatca

00000001 gtatcatctcctgaagcaacatctggttctacagg 00000035

<<<<<<<< ||||||||||||||||||||||||||||||||||| <<<<<<<<

38303438 gtatcatctcctgaagcaacatctggttctacagg 38303404

**Alignment of 117737_0258_2524 and chr17:39151355-39151389**

**117737_0258_2524 65 32 65 chr8 146274826 38324858 38324892**

acaaagggaa ttggtaacaa aacagaaata agtgtcaggg ggcaagacag 39151440

gcttataatt tctaaaccaa accagagaaa tgaaaagcca atgcagaata 39151390

TATCATCTCt TGAAGCAACA TCTGGTTCTA CAGGCtcagt agaaaagaag 39151340

caacagagaa gatcaattag aacttgttct gaatcagaga agtccactga 39151290

ggttgtgcca aagaagaaga taaaaaagga gcagg

00000002 tatcatctcctgaagcaacatctggttctacaggc 00000036

<<<<<<<< ||||||||| ||||||||||||||||||||||||| <<<<<<<<

39151389 tatcatctcttgaagcaacatctggttctacaggc 39151355

**Putative Deletion 2**

**Alignment of 008589_3095_1356 and chr15:42797244-42797307**

**008589_3095_1356 93 0 65 chr15 100338915 42797243 42797307**

ttaacaagct ttgagtgcaa gagattgaag agttcaaatc tgaccaagat 42797358

gttgatgttg gataagagaa ttctctgctc cccacctcta agttgccagc 42797308

CCTCCTAGAG CTACCTGTGG AGCAACCTGC TCAGATACAT CAAACATGGA 42797258

GACAGCACTC AAAGtagaat tataaagaag atcatgtcca tgttaacatt 42797208

attataaccc tacattttgt gcataaagtg taagtgtata agcatatcaa 42797158

tattaaaaag caag

00000001 cctcctagagctacctgtggagtcaacctgctcagatacatcaaacatgg 00000050

<<<<<<<< |||||||||||||||||||||| ||||||||||||||||||||||||||| <<<<<<<<

42797307 cctcctagagctacctgtggag.caacctgctcagatacatcaaacatgg 42797259

00000051 agacagcactcaaag 00000065

<<<<<<<< ||||||||||||||| <<<<<<<<

42797258 agacagcactcaaag 42797244

**Alignment of 008589_3095_1356 and chr15:42797390-42797426**

**008589_3095_1356 93 55 92 chr15 100338915 42797389 42797426**

acaggtagct ctaggagggc tggcaactta gaggtgggga gcagagaatt 42797339

ctcttatcca acatcaacat cttggtcaga tttgaactct tcaatctctt 42797389

GCACTCAAAG CTTGTTAAGA TAGTTAAGCG TGCATAAgtt aacttccaat 42797439

ttacatactc tgcttagaat ttgggggaaa atttagaaat ataattgaca 42797489

ggattattgg aaatttgtta taatgaatga aacattt

00000056 gcactcaaagcttgttaagatagttaagcgtgcataa 00000092

>>>>>>>> ||||||||||||||||||||||||||||||||||||| >>>>>>>>

42797390 gcactcaaagcttgttaagatagttaagcgtgcataa 42797426

**Rare isoform: validated by RT-PCR and Sanger sequencing: 6B**

## Alignment of 045624_1590_1179:Rare and chr22:21571798-21573177

**045624_1590_1179:Rare 57 1 60 102 98.4% 22 - 21571798 21573177 1380**

00000001 gagggggcagccttgggctgac 00000022

<<<<<<<< |||||||||||||||||||||| <<<<<<<<

21573177 gagggggcagccttgggctgac 21573156

00000023 ctaggacggtcagcttggtccctccgccgaagaccaca 00000060

<<<<<<<< ||||||||||||||||||||||||||||||| |||||| <<<<<<<<

21571835 ctaggacggtcagcttggtccctccgccgaataccaca 21571798

## Alignment of 045624_1590_1179:Rare and chr22:21060830-21060871

**045624_1590_1179:Rare 35 60 101 102 94.9% 22 - 21060830 21060871 42**

00000060 agcgctgttgtgccaaatcataca...atagtagtcagcctcatc 00000101

<<<<<<<< ||||||| |||||||||||||||| ||||| |||||||||| <<<<<<<<

21060871 agcgctgctgtgccaaatcatacagtaatagt...cagcctcatc 21060830
