# Supplementary material for: A transcriptional sketch of a primary human breast cancer by 454 deep sequencing
Source: BMC Genomics. 2009 Apr 20;10:163. doi: 10.1186/1471-2164-10-163 (PMC2678161; doi:10.1186/1471-2164-10-163)
Supplement: Additional file 5 — Biological validation of selected interesting transcripts. Word document containing the description of the RT-PCR validations for potential cancer-related transcripts identified in this study; the reanalysis of two Affymetrix and cDNA array breast cancer patients datasets for the investigation of the MALAT1 ncRNA expression pattern. [file 1471-2164-10-163-S5.doc]

## Additional File 5. Biological validations of selected interesting transcripts

## RT-PCR of fusions, novel or rare isoforms, new transcripts, putative deletion

Biological validation of in-silico predicted transcriptional categories by RT-PCR. Sample 1 is the RNA corresponding to the original breast cancer sample (1360), samples 2 and 3 correspond to RNA from two other lobular breast cancer tumor samples (1345 and 1645). The sequence identifiers, primers, transcript labels and the PCR product size are reported in the following tables. All the experiments have the Fermentas 50 bp MW marker, with the exception of ES2-ES3-HPRT1 which has the 100 bp Fermentas MW marker. C => COT DNA only, plus enzymes. HPRT1 => positive control, gene HPRT1. All the fourteen amplicons were validated by direct sequencing on the original lobular breast cancer cDNA library.


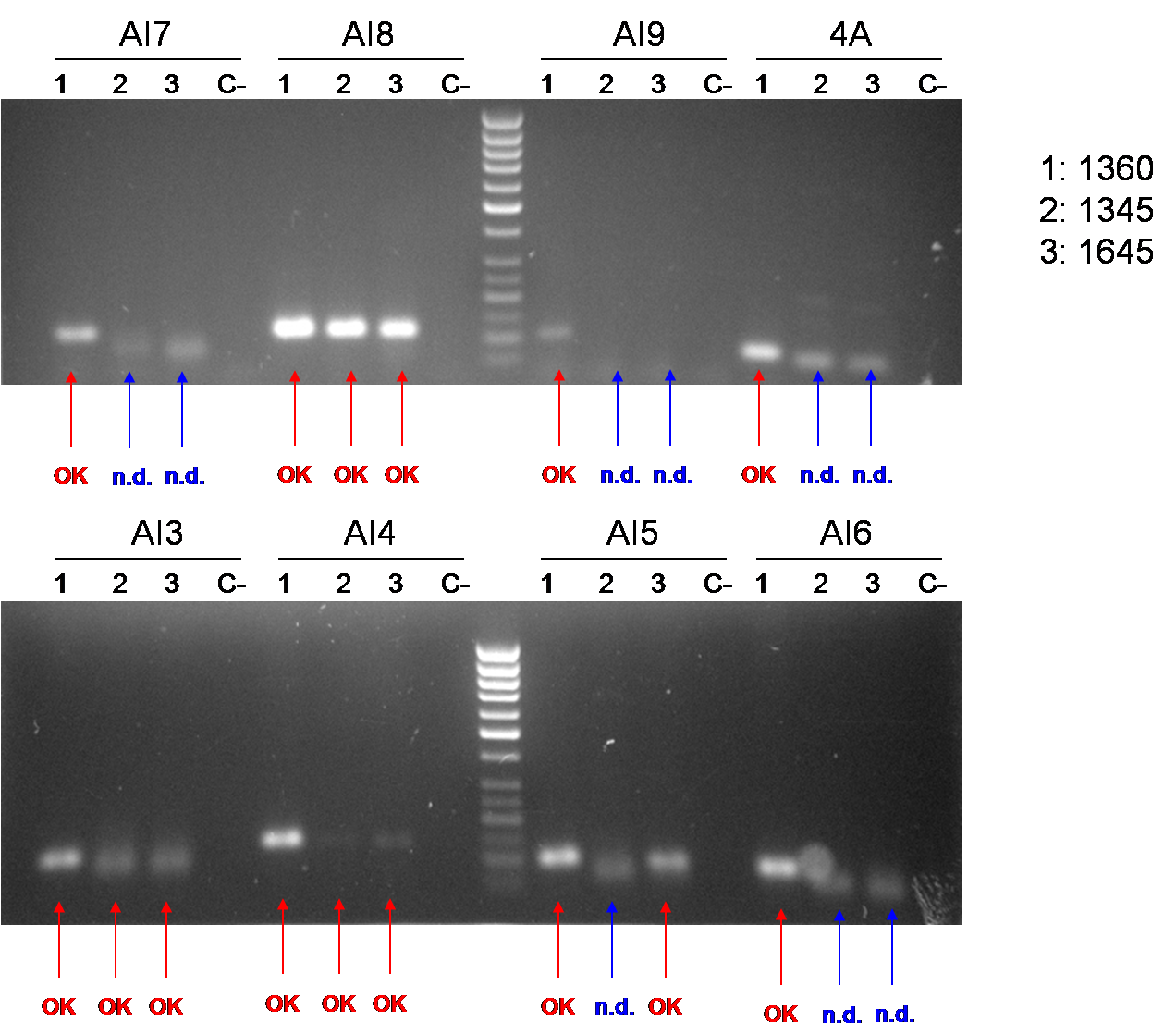


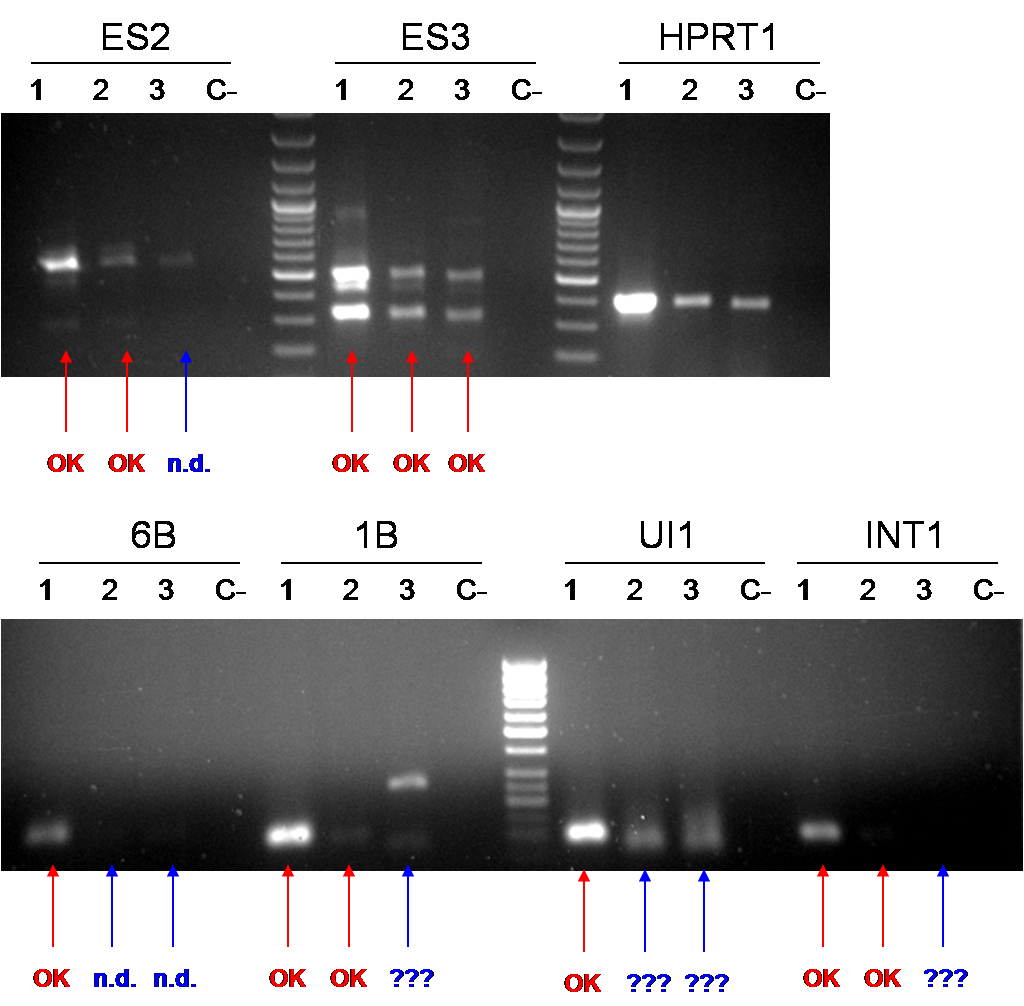


Further biological validation of *in-silico* predicted transcriptional categories by RT-PCR, selected from the 14 transcripts validated above. RNA from other six lobular breast cancer samples was subjected to RT-PCR. The primers, transcript labels and the PCR product size are reported in the following tables. All the experiments have the Fermentas 50 bp MW marker, with the exception of ES2-ES3-HPRT1 which has the 100 bp Fermentas MW marker. C => COT DNA only, plus enzymes. HPRT1 => positive control, gene HPRT1.


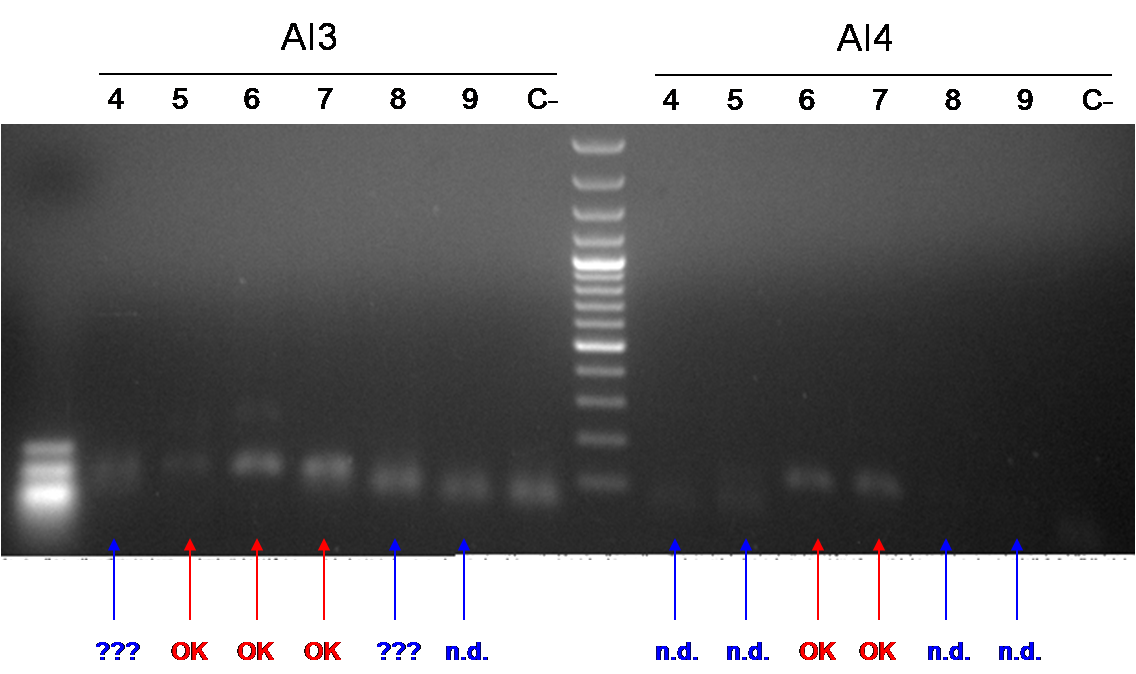


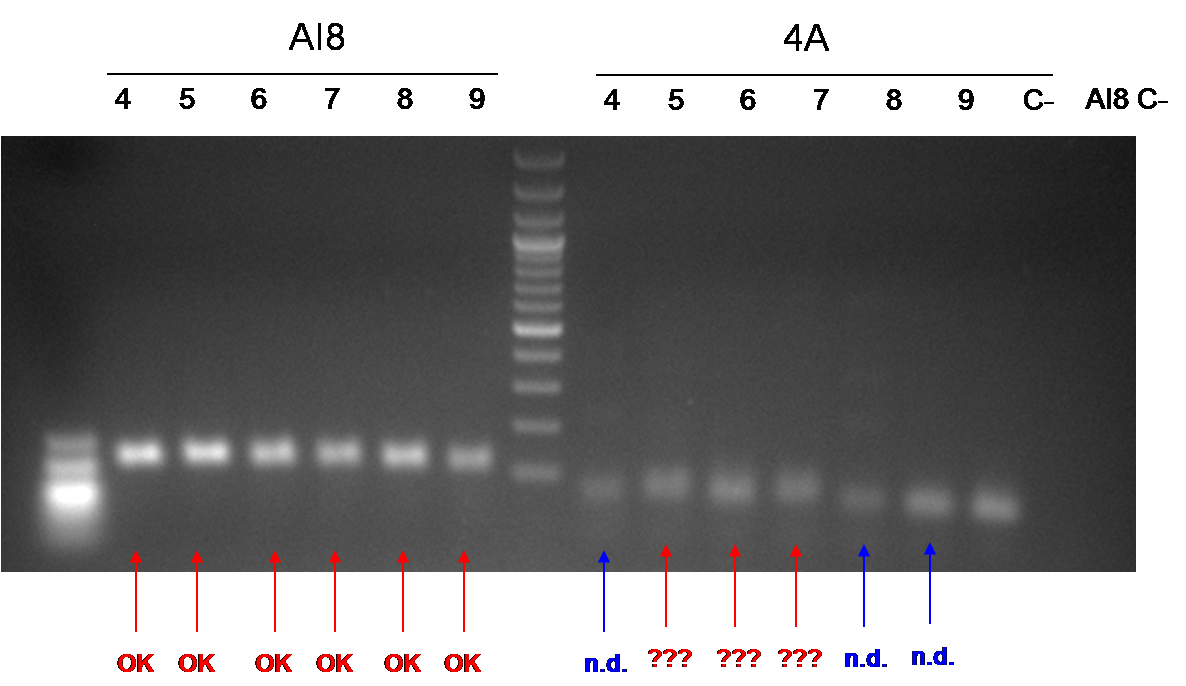


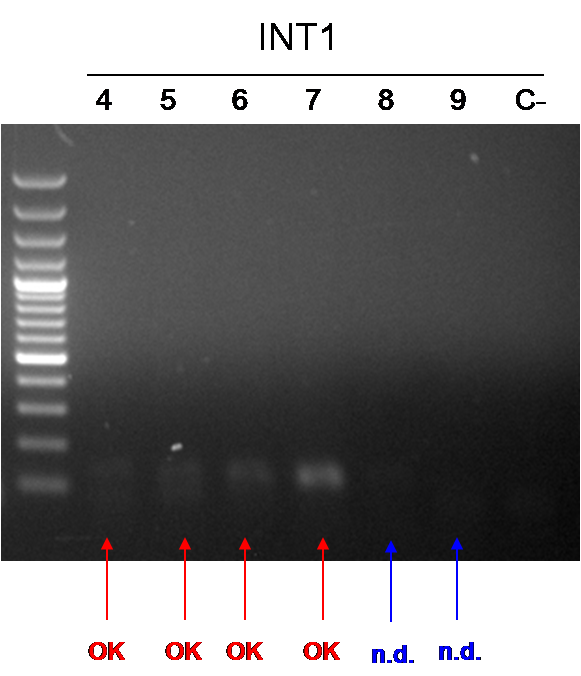


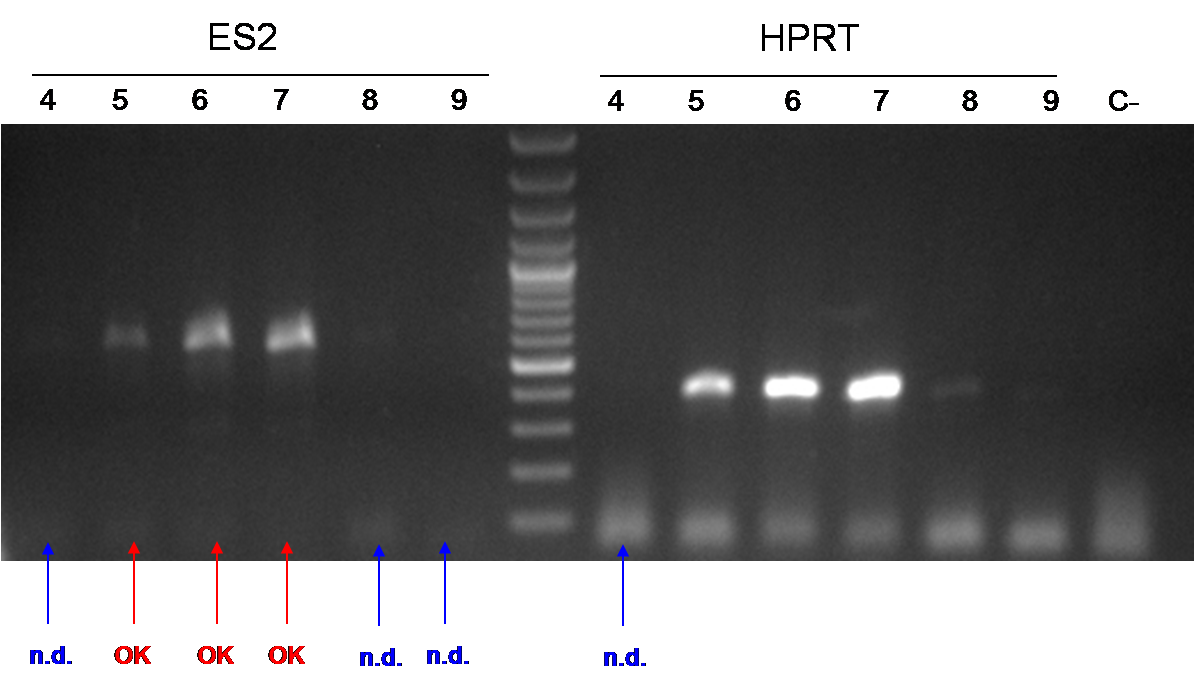


## Primer sequences for RT-PCR validation

Primer sequences and reaction conditions for RT-PCR validations of selected transcript categories.

| 1. ***Internal ID*** | 1. ***Read ID*** | 1. ***Primer For (5'=>3')*** | 1. ***Primer Rev (5'=>3')*** | 1. ***Ann T C*** |
| --- | --- | --- | --- | --- |
| 1. **Novel Isoforms: Exon Skipping** | | | | |
| 1. **ES2** | 1. **018640_1257_0905** | 1. **GCTGTTGGAGGAAAGGGA** | 1. **TAGGGATCGAGGCTTTGC** | 1. **57** |
| 1. **ES3** | 1. **057480_0973_1085** | 1. **GCTGTAGGGGAAAGTGCTA** | 1. **TGTGGATCTCTGGATGGCT** | 1. **57** |
| 1. **Novel Isoforms: New Splice Patterns** | | | | |
| 1. **AI3** | 1. **018086_0726_1326** | 1. **TGCATCACATCTGCATCGAG** | 1. **GTGTCTCTCTATACTTGACGG** | 1. **55** |
| 1. **AI4** | 1. **023612_1809_1670** | 1. **TCCTTCAGGCTATTCCCTCT** | 1. **GTGGGGACGGAATGATTAACAA** | 1. **59** |
| 1. **AI5** | 1. **239323_0568_3279** | 1. **CATGCTTGTCCACCCAGG** | 1. **CTTGGGAGACACAGGACTC** | 1. **59** |
| 1. **AI6** | 1. **017234_1267_3837** | 1. **GGCAACAACAATGCTGTATCC** | 1. **GTTATGCAGACACCTTCTGTTC** | 1. **55** |
| 1. **AI7** | 1. **082654_1071_3200** | 1. **ATTCTACTCCTAAGAATCTCCC** | 1. **CCCACCTGATGGTCATTC** | 1. **55** |
| 1. **AI8** | 1. **285069_1182_1741** | 1. **ACTGATTAAGTCAAACCCTCATTT** | 1. **ATTTCTGGCCTGGAGCTA** | 1. **55** |
| 1. **AI9** | 1. **011394_0819_2283** | 1. **TGTGGTTAGAGTTCTAAAGATACT** | 1. **TCTCATCGGCTATCGTTTGTA** | 1. **55** |
| 1. **Novel Transcripts: Unspliced Intronic** | | | | |
| 1. **UI1** | 1. **075828_1754_1488** | 1. **ACAACTCATCTGTTAGCAGC** | 1. **GGACACAGGTAATAGACTCTC** | 1. **55** |
| 1. **Novel Transcripts: Intergenic** | | | | |
| 1. **INT1** | 1. **006542_0430_2670** | 1. **TCCTCCTCTTTACCTTTGTGTT** | 1. **GGGAAATGTTACAAAATACTGTGTAC** | 1. **51** |
| 1. **Fusion transcripts (F), genome deletion (D) and rare isoform (I)** | | | | |
| 1. **4A (F)** | 1. **107781_1044_1738** | 1. **ACCTTCCTGTGGCTGAAG** | 1. **CCAATAGCGGCCAAGGTTAA** | 1. **58** |
| 1. **1B (D)** | 1. **167378_1645_3303** | 1. **GCAGAGTTTGTATCATCTCC** | 1. **CTCCAGCATCCTTAACTTTC** | 1. **55** |
| 1. **6B (I)** | 1. **045624_1590_1179** | 1. **AGCCTTGGGCTGACCTAG** | 1. **CGATGAGGCTGACTACTATTGT** | 1. **55** |

## Validation of selected transcripts

Summary of the validated reads divided into transcriptional categories. The size of the expected PCR product is in the last column.

| **Read ID** | **PCR product** | **Read ID** | **PCR product** |
| --- | --- | --- | --- |
| **Fusion transcript (F), deletion (D), rare isoform (I)** | | **New Isoforms: Exon Skipping** | |
| 4A (F) 107781_1044_1738 | 71bp | ES2 018640_1257_0905 | 271bp (standard isoform 528bp) |
| 1B (D) 167378_1645_3303 | 80bp | ES3 057480_0973_1085 | 312bp (standard isoform 480bp) |
| 6B (I) 045624_1590_1179 | 94bp | **New Isoforms: New Splice Patterns** | |
|  | | AI3 018086_0726_1326 | 90bp |
| **Intergenic Transcript** | | AI4 023612_1809_1670 | 119bp |
| INT1 006542_0430_2670 | 126bp | AI5 239323_0568_3279 | 91bp |
| **Unspliced Intronic Transcript** | | AI6 017234_1267_3837 | 97bp |
| UI1 075828_1754_1488 | 93bp | AI7 082654_1071_3200 | 93bp |
|  | | AI8 285069_1182_1741 | 99bp |
|  | | AI9 011394_0819_2283 | 98bp |

## Investigating *MALAT1* expression in breast cancer samples

A detailed meta-analysis of publicly available gene expression datasets from the CleanEx database (http://www.cleanex.isb-sib.ch, Praz 2004) revealed nine breast cancer gene expression datasets suitable for MALAT1 expression analysis. Four of these datasets were composed of large, well characterized and stratified cohorts of patients and included control and Tamoxifen treatment regimes (GSE6532B, GSE4922B, GSE3494B, GSE1456B). When the normalized intensity of the probes corresponding to MALAT1 was analyzed we detected variable expression, but also observed a significant density of intensities above the normalized mean value. We selected a subset of 137 ER+ breast cancer patients (untreated) from the GSE6532B dataset (Loi 2007) and identified the data points derived from the 10 Affymetrix HG-U133B probesets corresponding to MALAT1. Unsupervised clustering revealed a wide range of expression of this transcript in this experiment, with good agreement between expression values from probesets in different samples, as shown in **Fig. 1**.


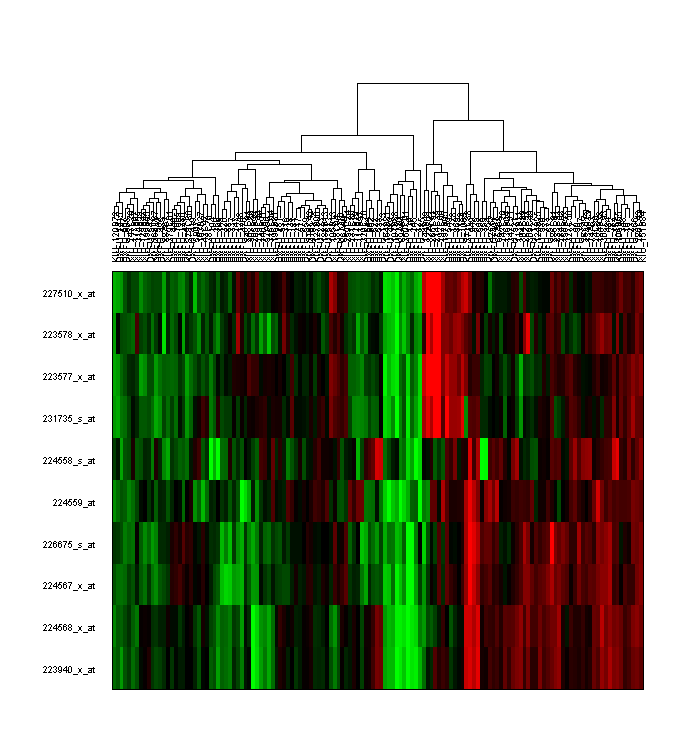


**Fig. 1** – unsupervised clustering of *MALAT1* Affymetrix array expression values in the Tamoxifen untreated subset (LOI dataset) of sample GSE6532B shows a wide range of variability and coherence between the different probeset expression values

In **Fig.2** we plot the bins of the log2 normalized Affymetrix expression values (each point is the mean of the values of the 10 probesets) for the *MALATI* ncRNA from the 137 ER+ breast cancer patients (not treated with Tamoxifen) in GSE6532B. This plot reveals a wide range of gene expression values, with a number of highly expressed outliers. The Coefficient of Variation is 42.7 %.

**Fig. 2** – plot of *MALAT1* frequency of log2 normalized Affymetrix intensity values in the Tamoxifen untreated subset of ER+ breast cancer patients sample GSE6532B (LOI untreated dataset). N=137, CV=42.7%. The Y axis values represent the counts of the subjects in each bin.

In **Fig.3** we plot the bins of log2 normalized Affymetrix expression values (mean of the 10 probesets) of *MALATI* for the 277 ER+ breast cancer patients treated with Tamoxifen in GSE6532B. Comparing with patients who were not treated with Tamoxifen, we find a larger variation in expression, which is reflected in a Coefficient of Variation of 83.9 %. This observation suggests that Tamoxifen treatment may have an effect on *MALATI* expression.

**Fig. 3** – plot of *MALAT1* frequency of log2 normalized Affymetrix intensity values in the Tamoxifen treated subset of ER+ breast cancer patients sample GSE6532B (LOI Tam-treated dataset). N=277, CV=83.9%. The Y axis values represent the counts of the subjects in each bin.

Finally, we investigated *MALAT1* expression in a cDNA array dataset (TAM113 dataset) which probed total polyA+ RNA from 113 ER+ lobular and ductal breast cancer samples, treated with Tamoxifen (**Fig. 4**) (Loi 2008). Although this is a completely different platform, we found that, similar to Tamoxifen treated samples queried on Affymetrix arrays, MALAT1 expression showed a wide range of expression values, with multiple patient samples sitting well outside the core density of the distribution, and a CV of 67% (Fig. 3)

Fig. 4 – plot of *MALAT1* frequency of log2 normalized cDNA array intensity values in the Tamoxifen treated ER+ breast cancer patients TAM113 dataset. N=113, CV=67%.

These results from both Tamoxifen treated and untreated ER+ breast cancer patients with two different microarray technologies, reinforce our sequence-based findings that high *MALAT1* expression may be episodically associated with single breast tumours. It is likely that the sensitivity of our deep sequencing approach facilitated the detection of this ncRNA in our sample. We also note that the range of gene expression values (reflected in the Coefficients of Variation) is higher in Tamoxifen-treated versus Tamoxifen-untreated breast cancer samples.

Praz 2004:

Praz V, Jagannathan V, Bucher P. ‘CleanEx: a database of heterogeneous gene expression data based on a consistent gene nomenclature’. *Nucleic Acids Res.* 2004 Jan 1;32:D542-7.

Loi 2007:

Loi S, Haibe-Kains B, Desmedt C, Lallemand F, Tutt AM, Gillet C, Ellis P, Harris A, Bergh J, Foekens JA, Klijn JG, Larsimont D, Buyse M, Bontempi G, Delorenzi M, Piccart MJ, Sotiriou C. ‘Definition of clinically distinct molecular subtypes in estrogen receptor-positive breast carcinomas through genomic grade’**.** *J Clin Oncol*. 2007 Apr 1;25(10):1239-46.

Loi 2008:

Loi S, Haibe-Kains B, Desmedt C, Wirapati P, Lallemand F, Tutt AM, Gillet C, Ellis P, Ryder K, Reid JF, Daidone MG, Pierotti MA, Berns EM, Jansen MP, Foekens JA, Delorenzi M, Bontempi G, Piccart MJ, Sotiriou C. ‘Predicting prognosis using molecular profiling in estrogen receptor-positive breast cancer treated with tamoxifen’**.** *BMC Genomics*. 2008 May 22;9:239.
